# Supplementary material for: Global diversity dynamics in the fossil record are regionally heterogeneous
Source: Nat Commun. 2022 May 18;13:2751. doi: 10.1038/s41467-022-30507-0 (PMC9117201; doi:10.1038/s41467-022-30507-0)
Supplement: Supplementary file 1 — Supplementary Information [file 41467_2022_30507_MOESM1_ESM.pdf]

Supplementary Figures 1–7. Spatial extent of fossil occurrence data in each sampling region under each spatial standardisation treatment. Treatments cover no standardisation, standardisation of longitude-latitude (lng-lat) extent, standardisation of minimum spanning tree length (MST), and standardisation by both methods. Prior to any form of standardisation, each region shows significant variation in the spatial extent of its data, but standardisation by minimum spanning tree length can generally be effectively applied in each case. The displayed longitude and latitude distributions are the same when both standardisation methods are employed as longitude-latitude standardisation is applied prior to minimum spanning tree standardisation. Individual captions which detail the region and data standardisation treatment are embedded in each plot. Source data are provided as a Source Data file.

Supplementary Figures 8–14. Sampling-corrected diversity estimated at stage level using shareholder quorum subsampling (SQS), under each spatial standardisation treatment, for each study region. Variation between diversity trajectories at each quorum level (q) are small, following the expected pattern of geometric similarity between estimates at different levels of sampling coverage. Treatments cover no standardisation, standardisation of longitude-latitude (lng-lat) extent, standardisation of minimum spanning tree length (MST), and standardisation by both methods. Individual captions which detail the region and data standardisation treatment are embedded in each plot. 95% analytical confidence intervals are displayed for each sampling-corrected diversity trajectory. Source data are provided as a Source Data file.

Supplementary Figures 15–35. Probabilistic extinction, speciation and net diversification rates estimated by PyRate and probabilistic sampling-corrected diversity estimated using mcmcDivE, for each sampling region under each data standardisation treatment. Extinction and speciation histograms positively (log Bayes Factor [BF] = 2) or strongly (log BF = 6) support rate shifts in each study region throughout the Late Permian to Early Jurassic, but the timing of these shifts and their magnitudes vary strongly between regions, and are also affected by spatial sampling bias, highlighting the need for correction of this bias when estimating diversification dynamics from fossil occurrence data. Treatments cover no standardisation, standardisation of longitude-latitude (lng-lat) extent, standardisation of minimum spanning tree length (MST), and standardisation by both methods. 50%, 75% and 95% highest posterior densities (HPD) are displayed for each probabilistic rate estimate.

Individual captions which detail the region and data standardisation treatment are embedded in each plot. Source data are provided as a Source Data file.

Supplementary Figures 37–43. Comparison of probabilistic origination, extinction and origination rates, and diversity under each data standardisation treatment for each sampling region. Variation between rate and diversity curves under each spatial standardisation treatment highlight the need for correction of spatial sampling bias when estimating diversification dynamics from fossil occurrence data. The broad degree of similarity through time between the rate curves, however, suggests that they are slightly more robust to spatial sampling bias compared to diversity from bin to bin. Treatments cover no standardisation, standardisation of longitude-latitude (lng-lat) extent, standardisation of minimum spanning tree length (MST), and standardisation by both methods. 50%, 75% and 95% highest posterior densities (HPD) are displayed for each probabilistic rate estimate. Individual captions which detail the region and data standardisation treatment are embedded in each plot. Source data are provided as a Source Data file.

Supplementary Figures 44. Bin-wise occurrence age uncertainty distributions in the stratigraphically revised, composite dataset. Relative to their parent bin duration, fossil occurrence ages are generally more precise, highlighting the improved age resolution in our dataset. Boxplots display the range, 1st and 3rd quartiles and median for each set of fossil occurrence age uncertainties. WUC = Wuchiapingian, CHX = Changhsingian, IND = Induan, OLE = Olenekian, ANS = Anisian, LAD = Ladinian, CRN = Carnian, Nor = Norian, RHT = Rhaetian, Het = Hettangian.

Supplementary Tables 1–64. Correlation tests between spatial extent and sampling-standardised diversity under each data standardisation treatment for each sampling region. Correlation tests were performed with diversity at standardisation thresholds of 40, 50, 60 and 70%. Spatial extent is measured using minimum spanning tree length, latitude range and longitude range. Treatments cover no standardisation, standardisation of longitude-latitude extent (lng-lat), standardisation of minimum spanning tree (MST) length, and standardisation by both methods. Supplementary Tables 1–32 record Pearson correlations and Supplementary Tables 33–64 record Spearman correlations.

Supplementary Table 65. Settings of birth-death-preservation simulations of the mcmcDivE model. 100 datasets were generated and run from 35 time units in the past (e.g. millions of years) under each setting, before estimation of original diversity from the sampled diversity in each dataset using traditional methods and mcmcDivE.

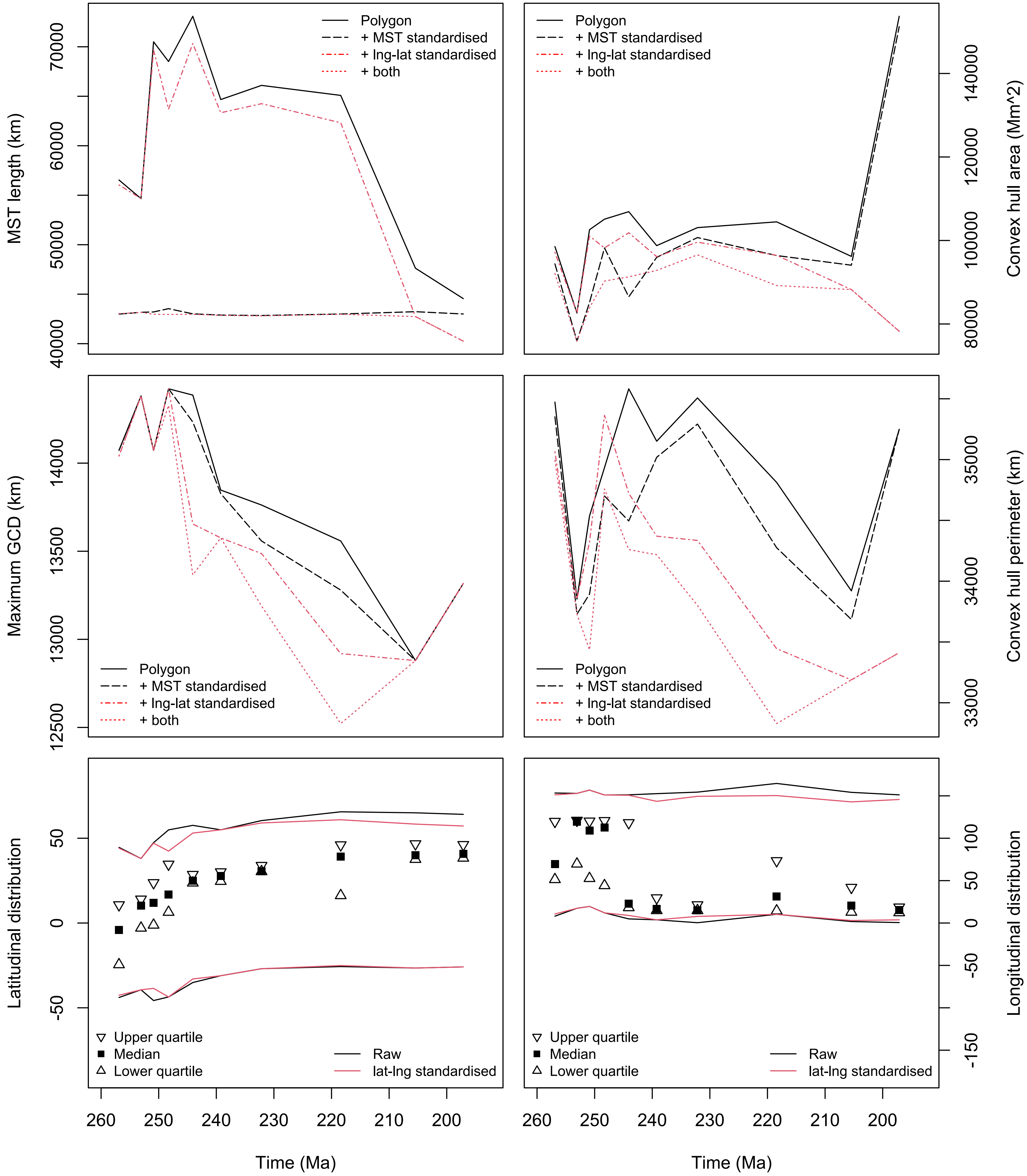

Supplementary Figure 1. Spatial properties of the Circumtethys region under each data standardisation treatment

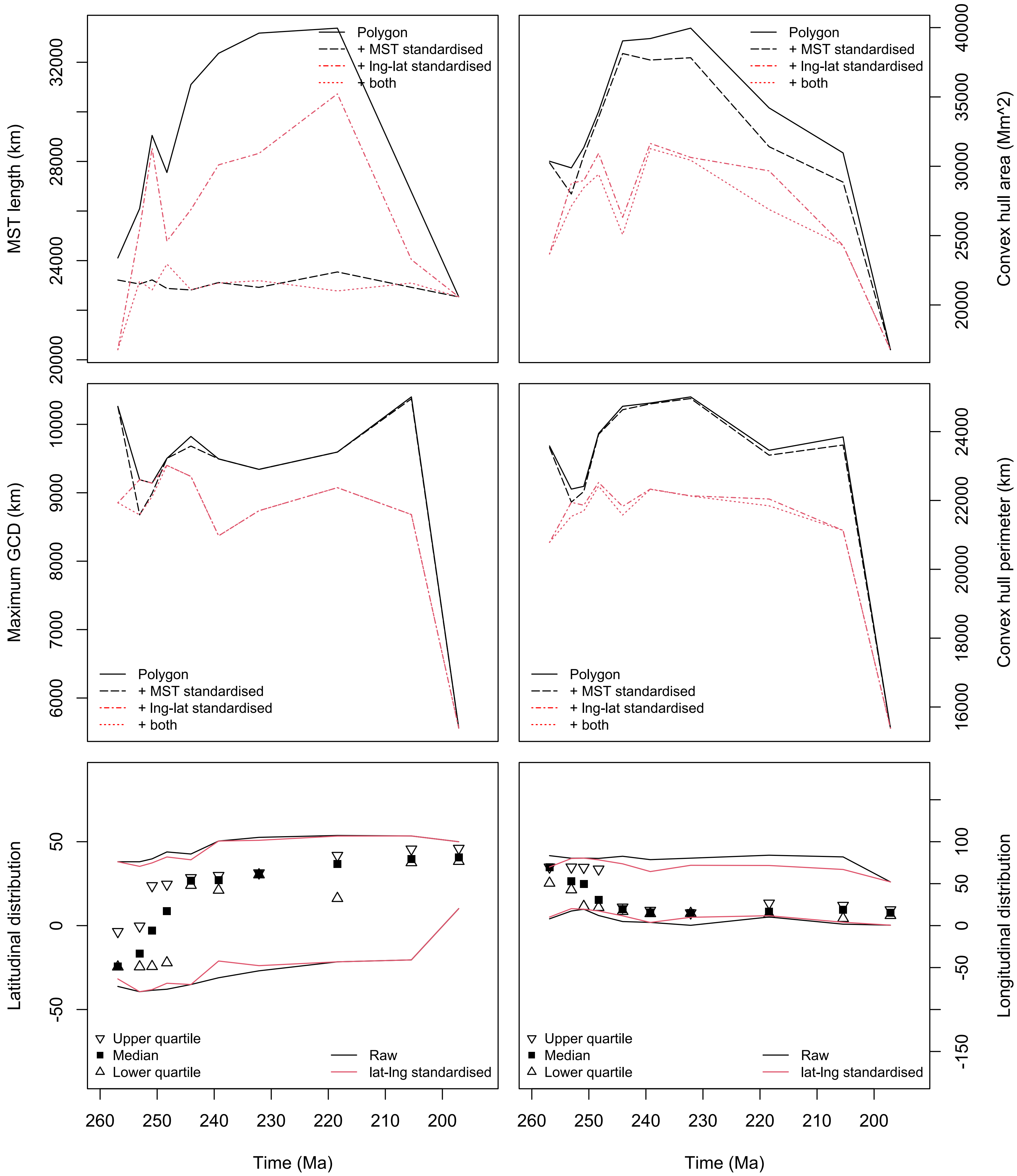

Supplementary Figure 2. Spatial properties of the West Circumtethys region under each data standardisation treatment

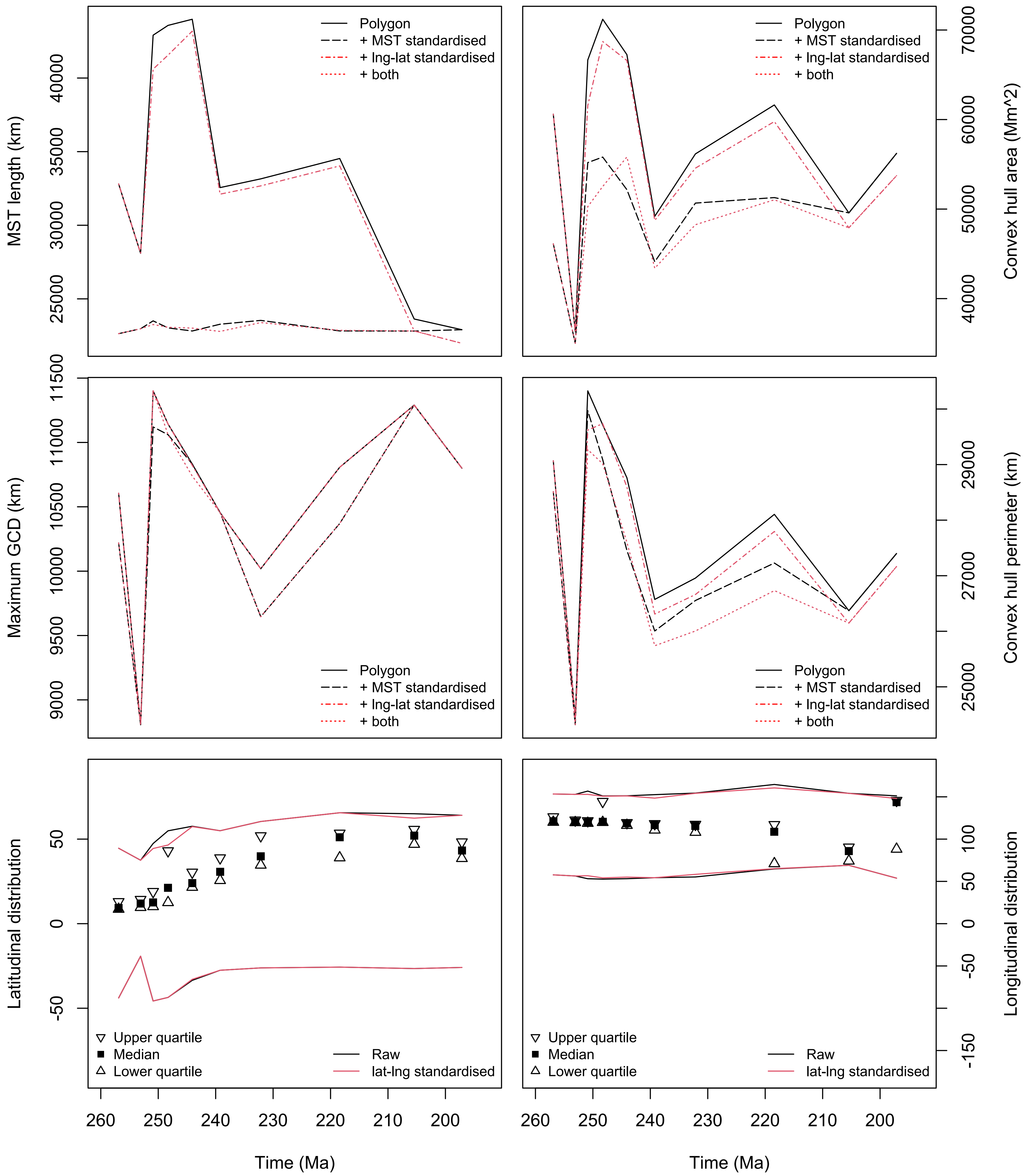

Supplementary Figure 3. Spatial properties of the East Circumtethys region under each data standardisation treatment

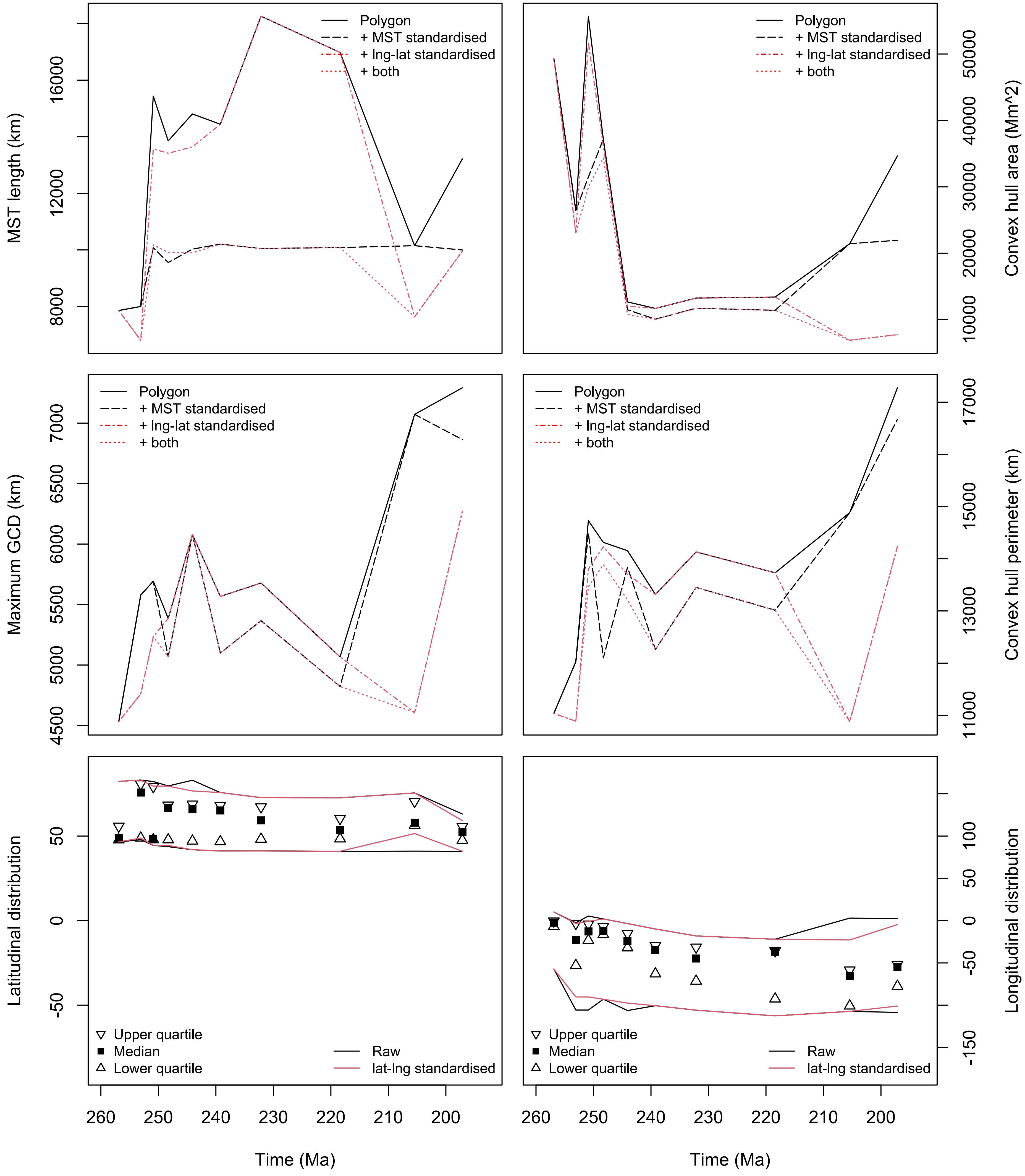

Supplementary Figure 4. Spatial properties of the Boreal region under each data standardisation treatment

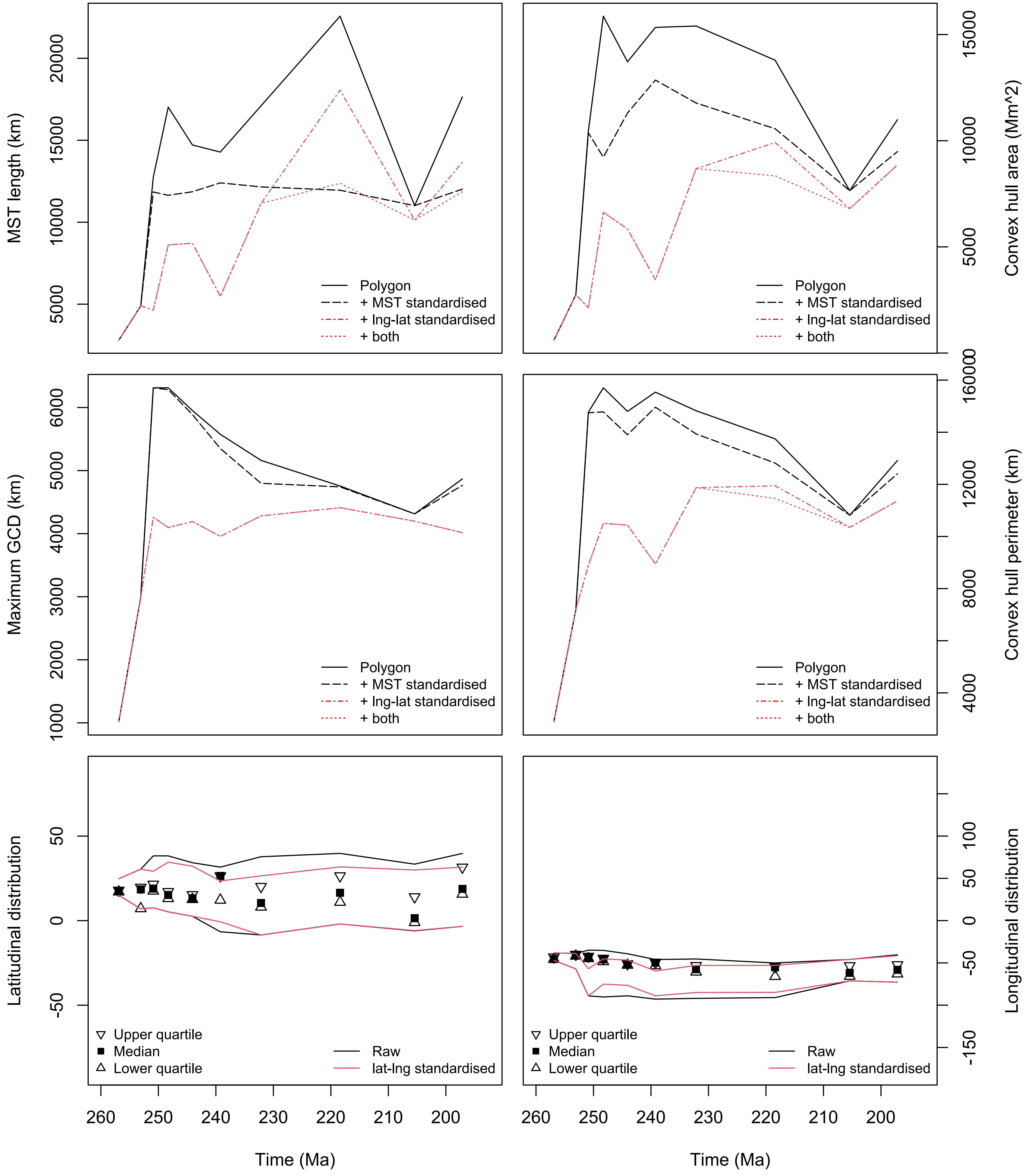

Supplementary Figure 5. Spatial properties of the North Panthalassic region under each data standardisation treatment

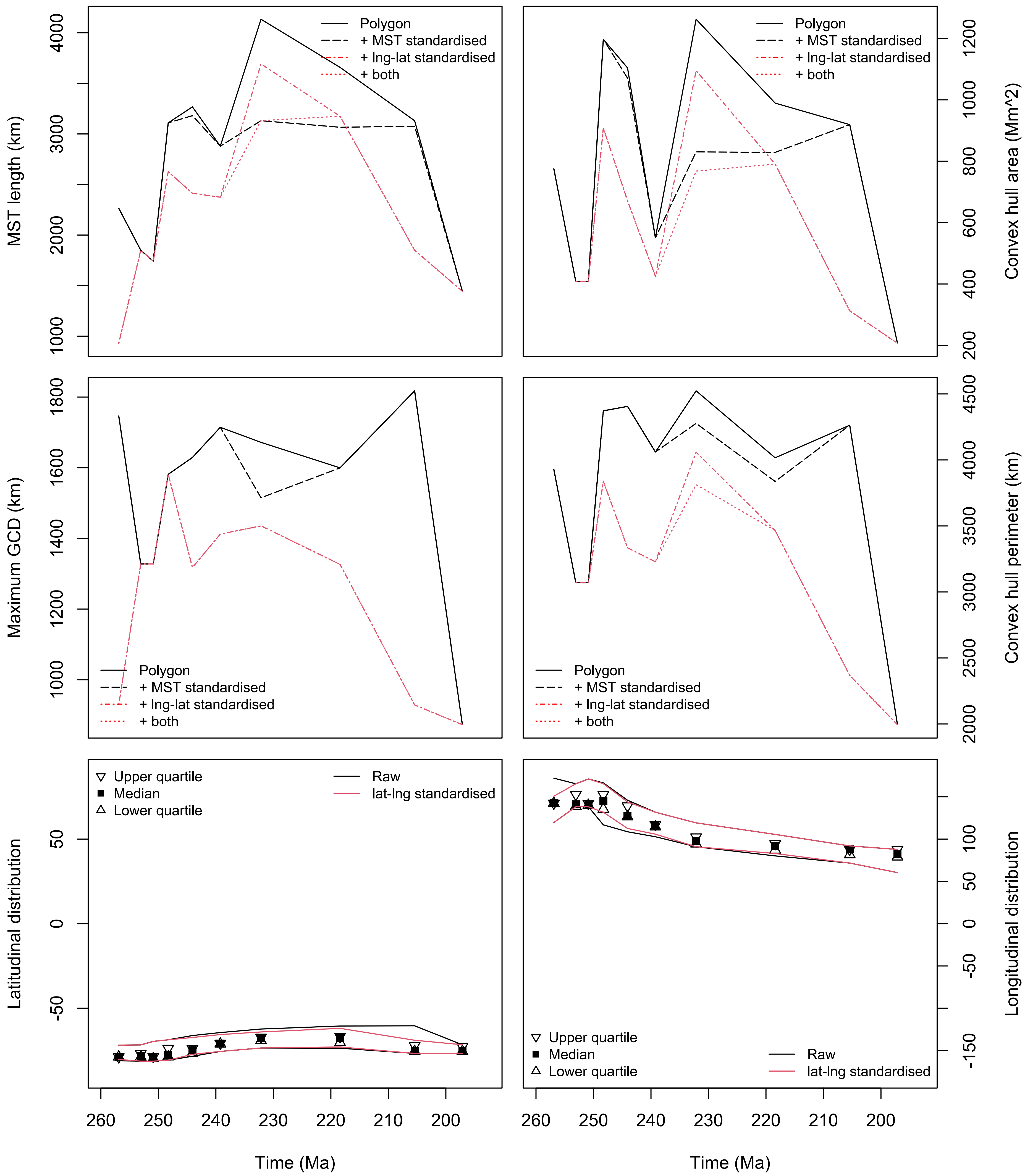

Supplementary Figure 6. Spatial properties of the Tangaroan region under each data standardisation treatment

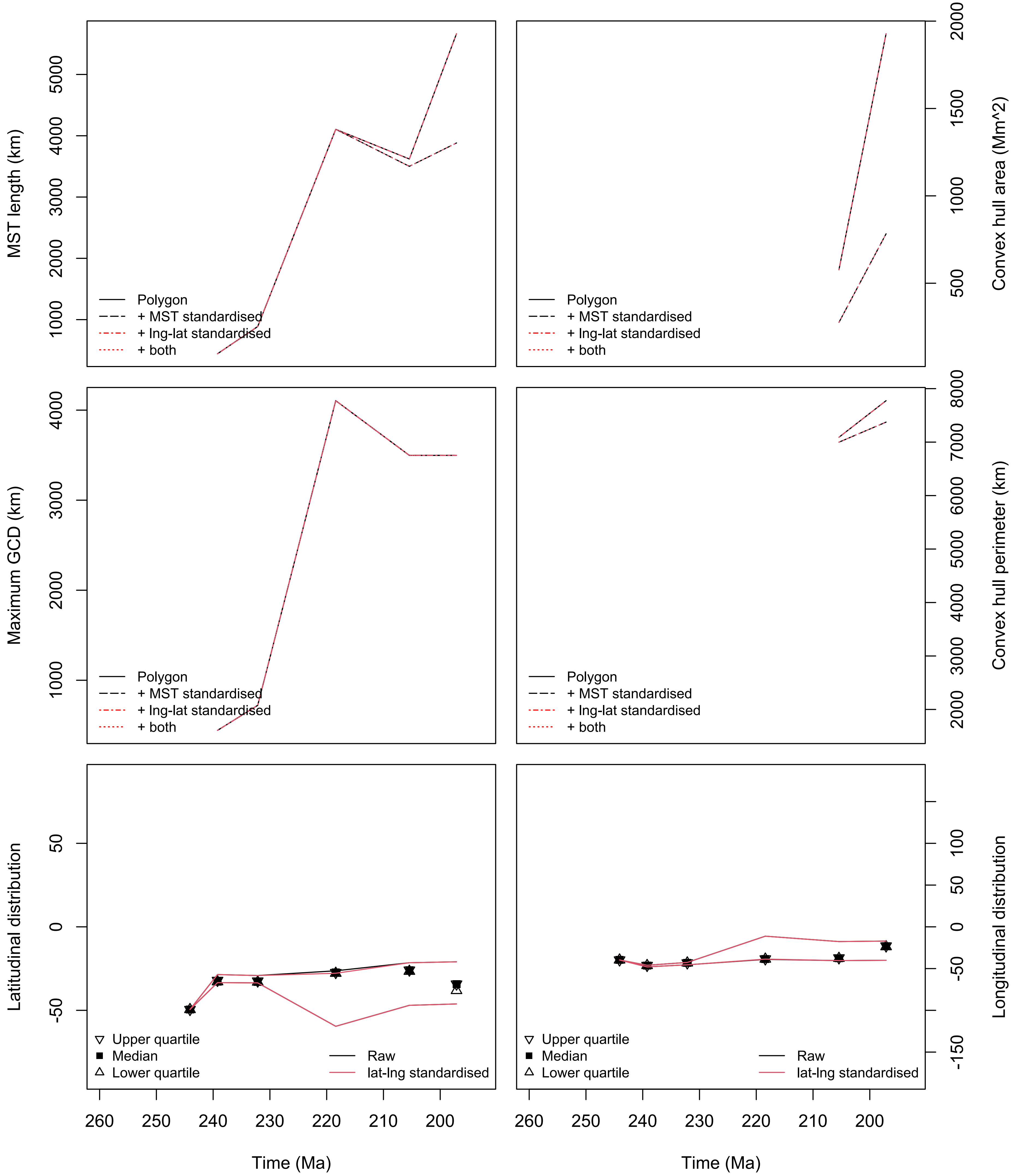

Supplementary Figure 7. Spatial properties of the South Panthalassic region under each data standardisation treatment

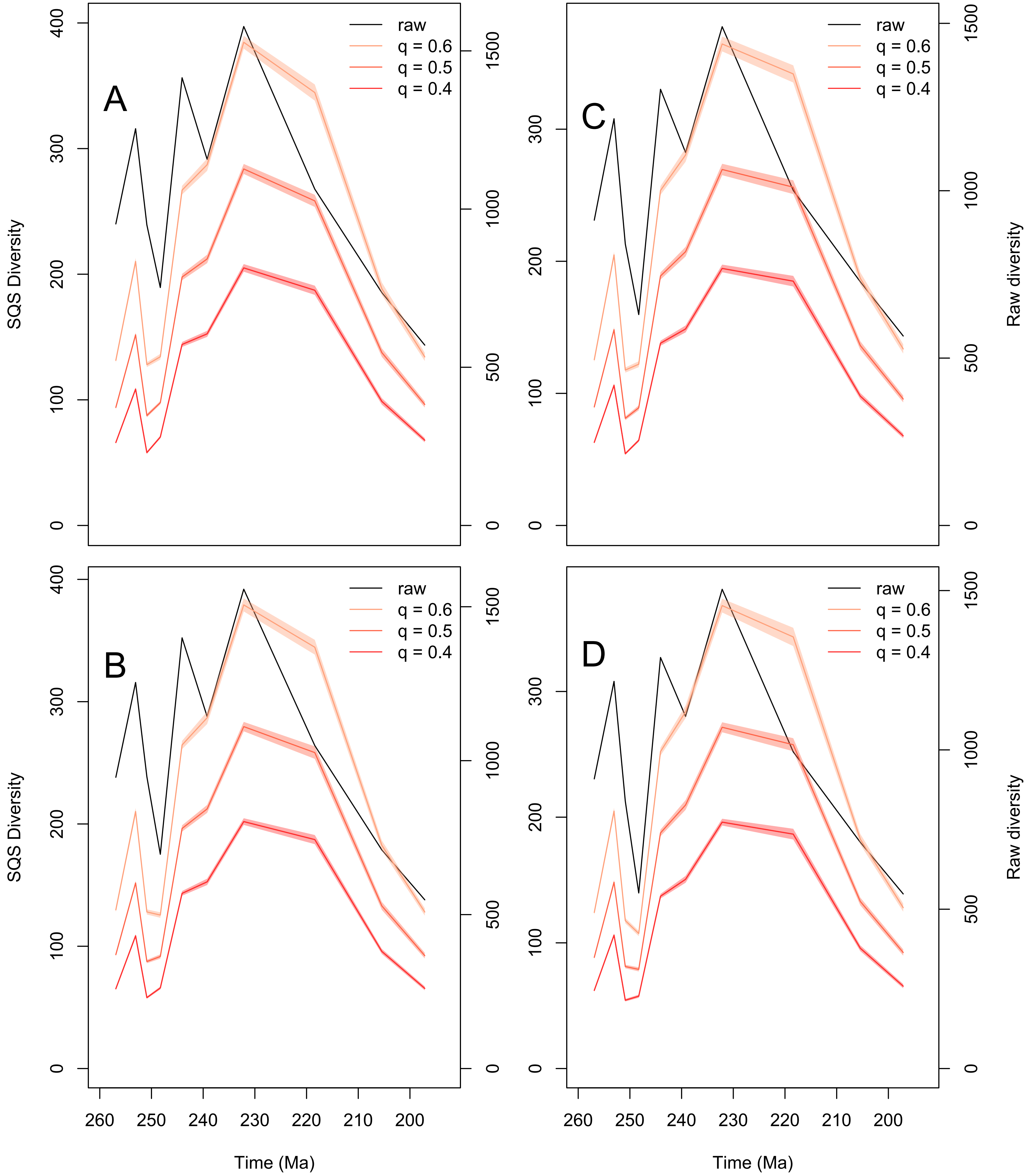

upplementary Figure 8. Stage-wise sampling-standardised diversity for the Circumtethys region under each data standardisation treatme
(A) Unstandardised. (B) MST standardisation. (C) Longitude-latitude standardisation. (D) MST + longitude-latitude standardisation.

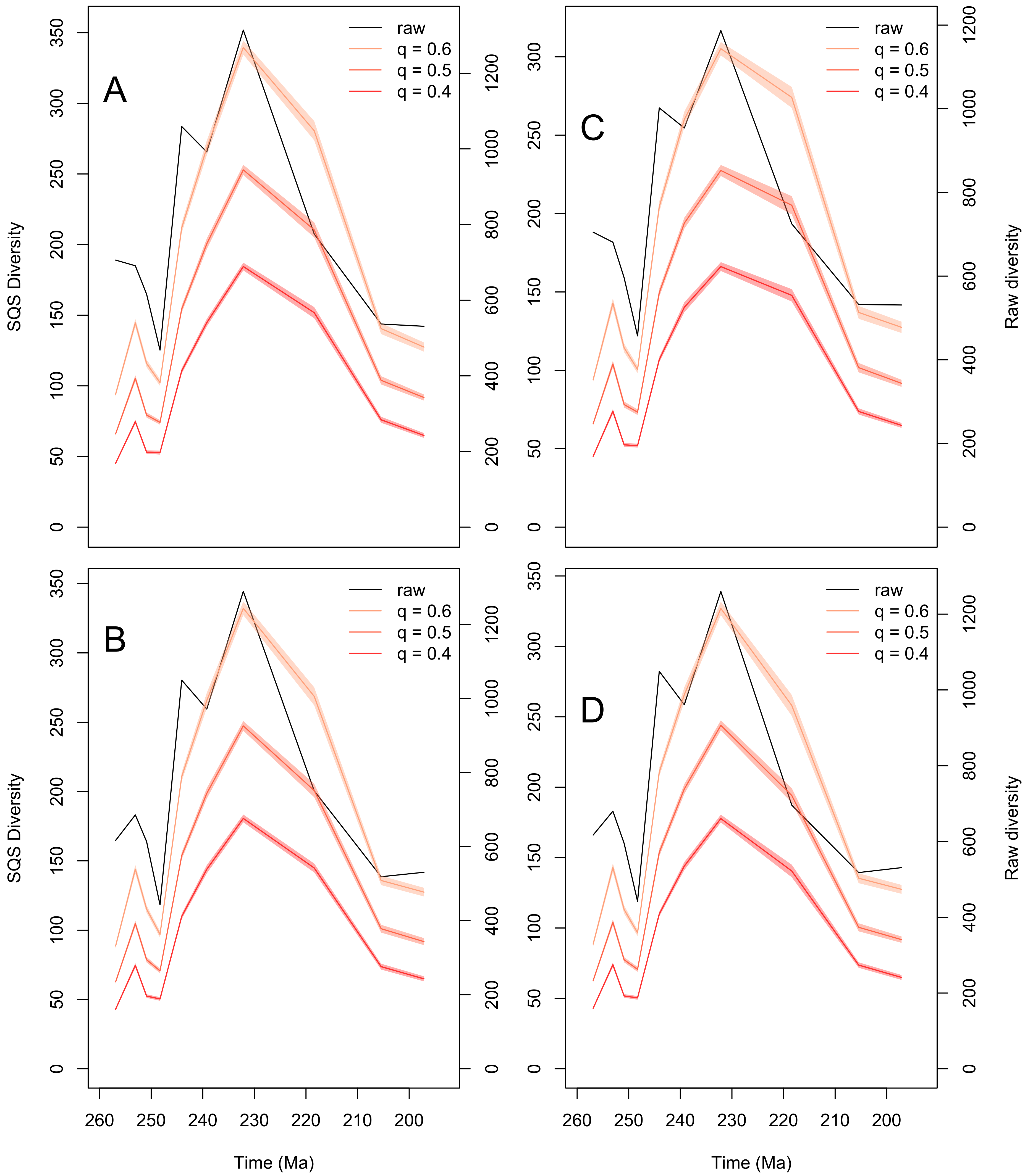

lementary Figure 9. Stage-wise sampling-standardised diversity for the West Circumtethys region under each data standardisation treatr
(A) Unstandardised. (B) MST standardisation. (C) Longitude-latitude standardisation. (D) MST + longitude-latitude standardisation.

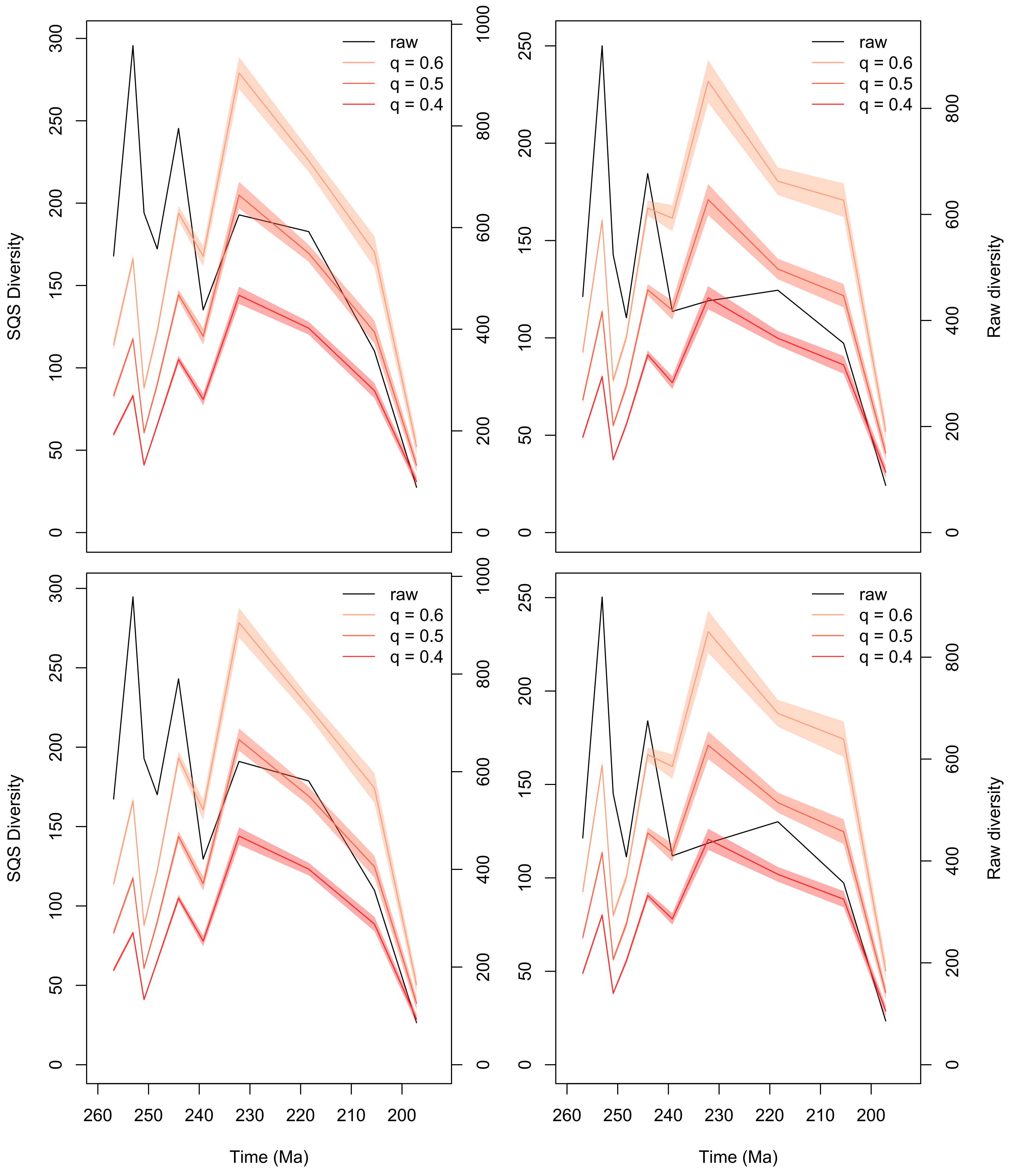

lementary Figure 10. Stage-wise sampling-standardised diversity for the East Circumtethys region under each data standardisation treat
(A) Unstandardised. (B) MST standardisation. (C) Longitude-latitude standardisation. (D) MST + longitude-latitude standardisation.

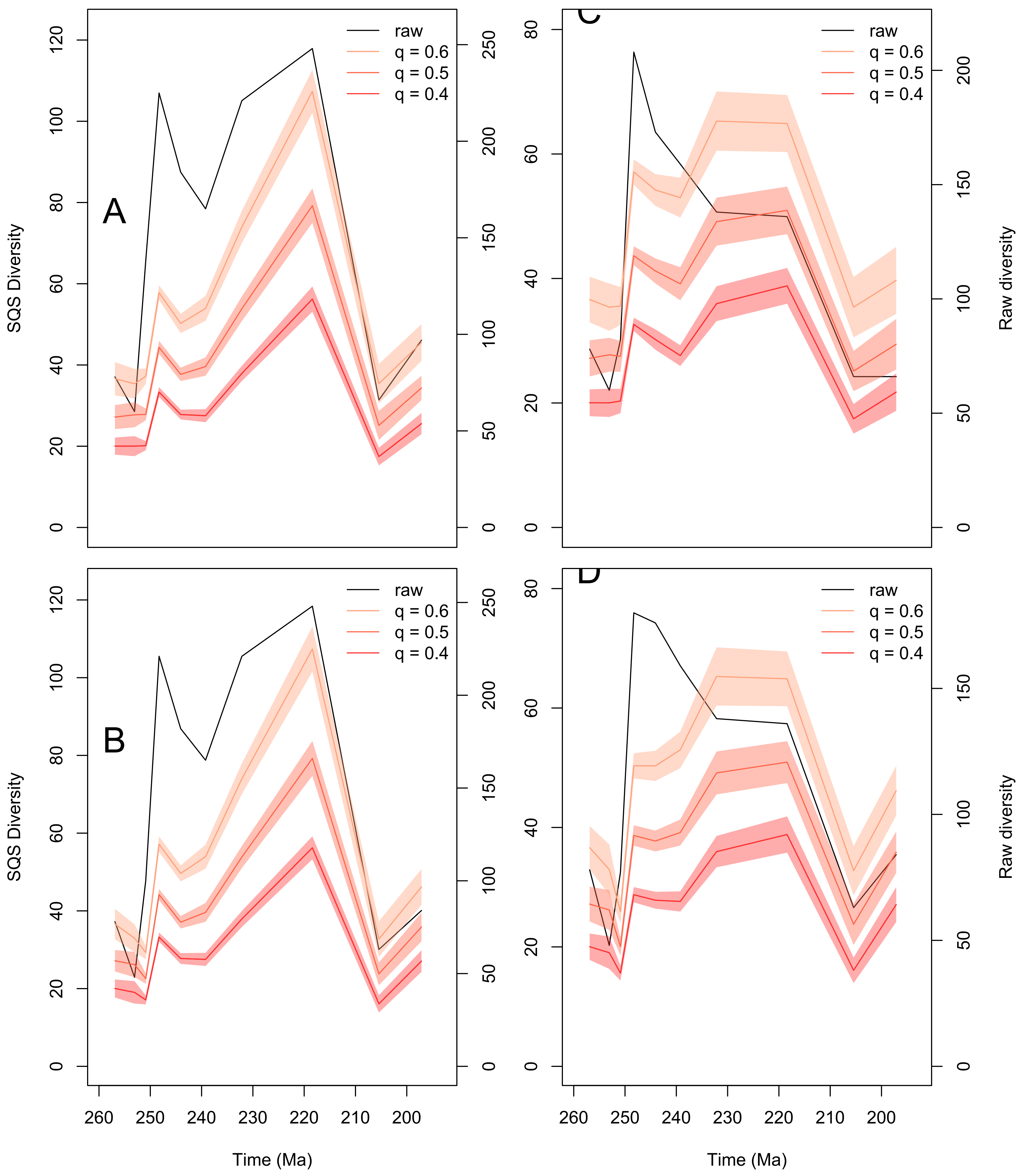

Supplementary Figure 11. Stage-wise sampling-standardised diversity for the Boreal region under each data standardisation treatment. (A) Unstandardised. (B) MST standardisation. (C) Longitude-latitude standardisation. (D) MST + longitude-latitude standardisation.

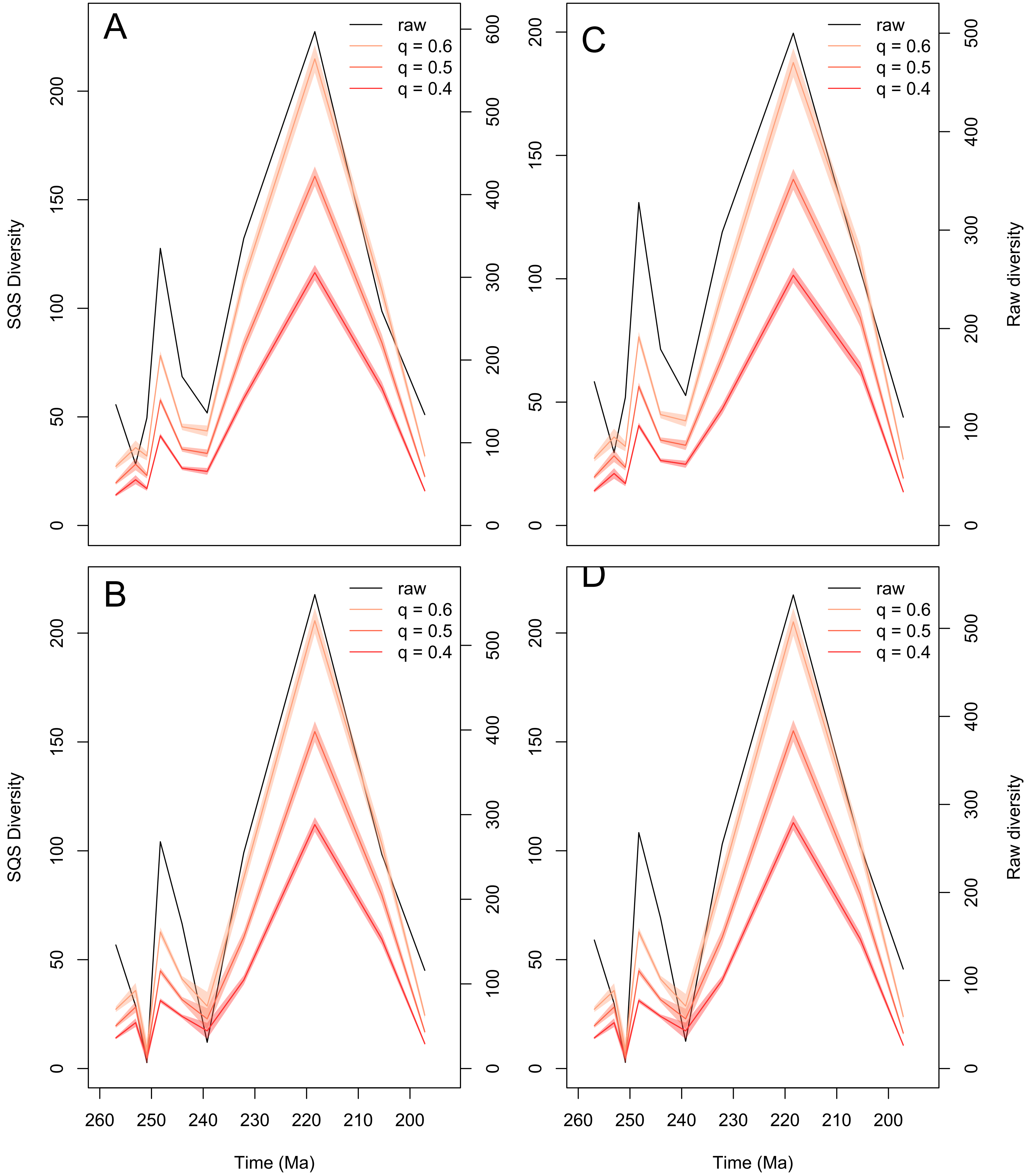

ementary Figure 12. Stage-wise sampling-standardised diversity for the North Panthalassic region under each data standardisation treat
(A) Unstandardised. (B) MST standardisation. (C) Longitude-latitude standardisation. (D) MST + longitude-latitude standardisation.

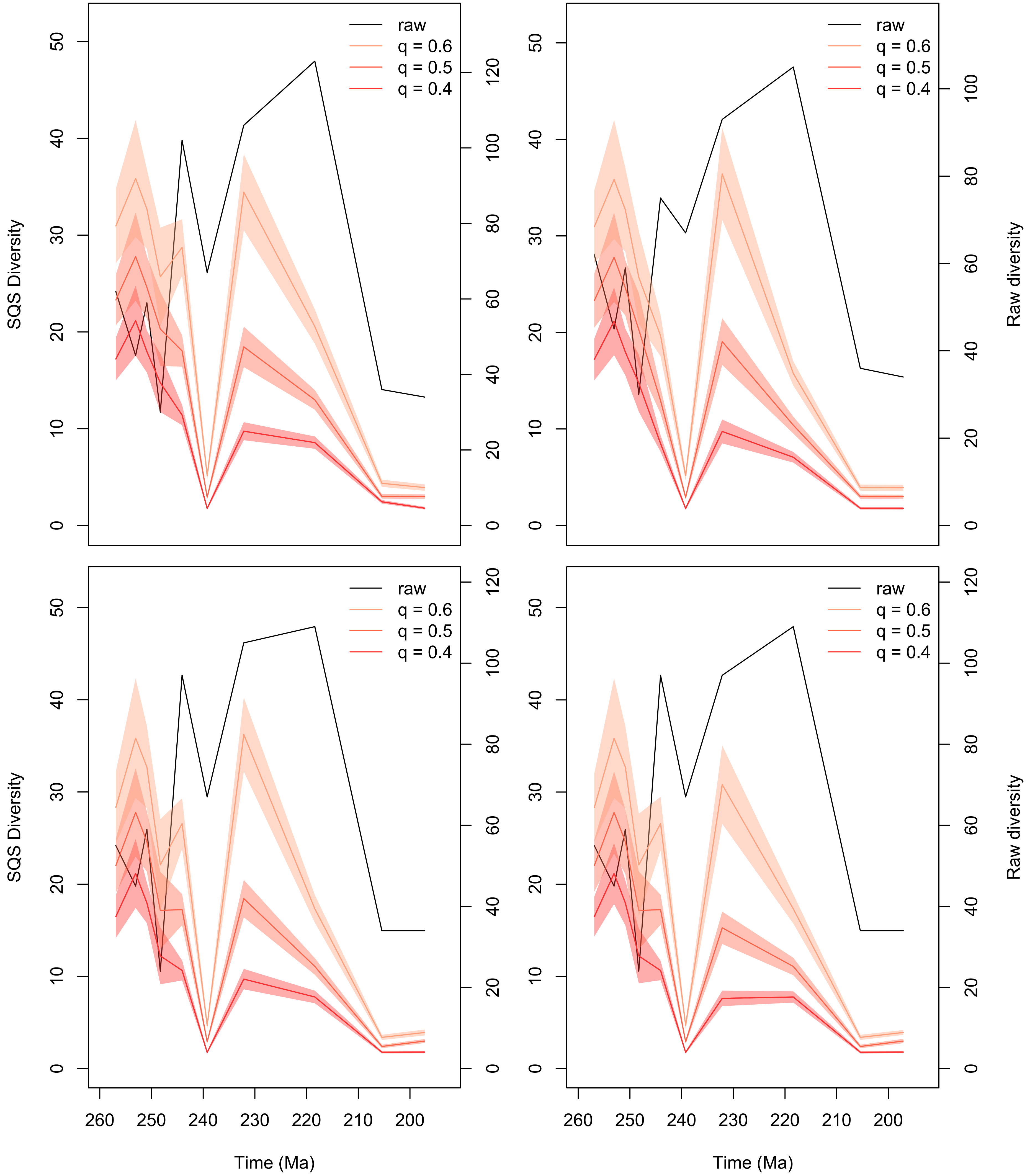

[illegible]upplementary Figure 13. Stage-wise sampling-standardised diversity for the Tangaroan region under each data standardisation treatmen[illegible]
(A) Unstandardised. (B) MST standardisation. (C) Longitude-latitude standardisation. (D) MST + longitude-latitude standardisation.

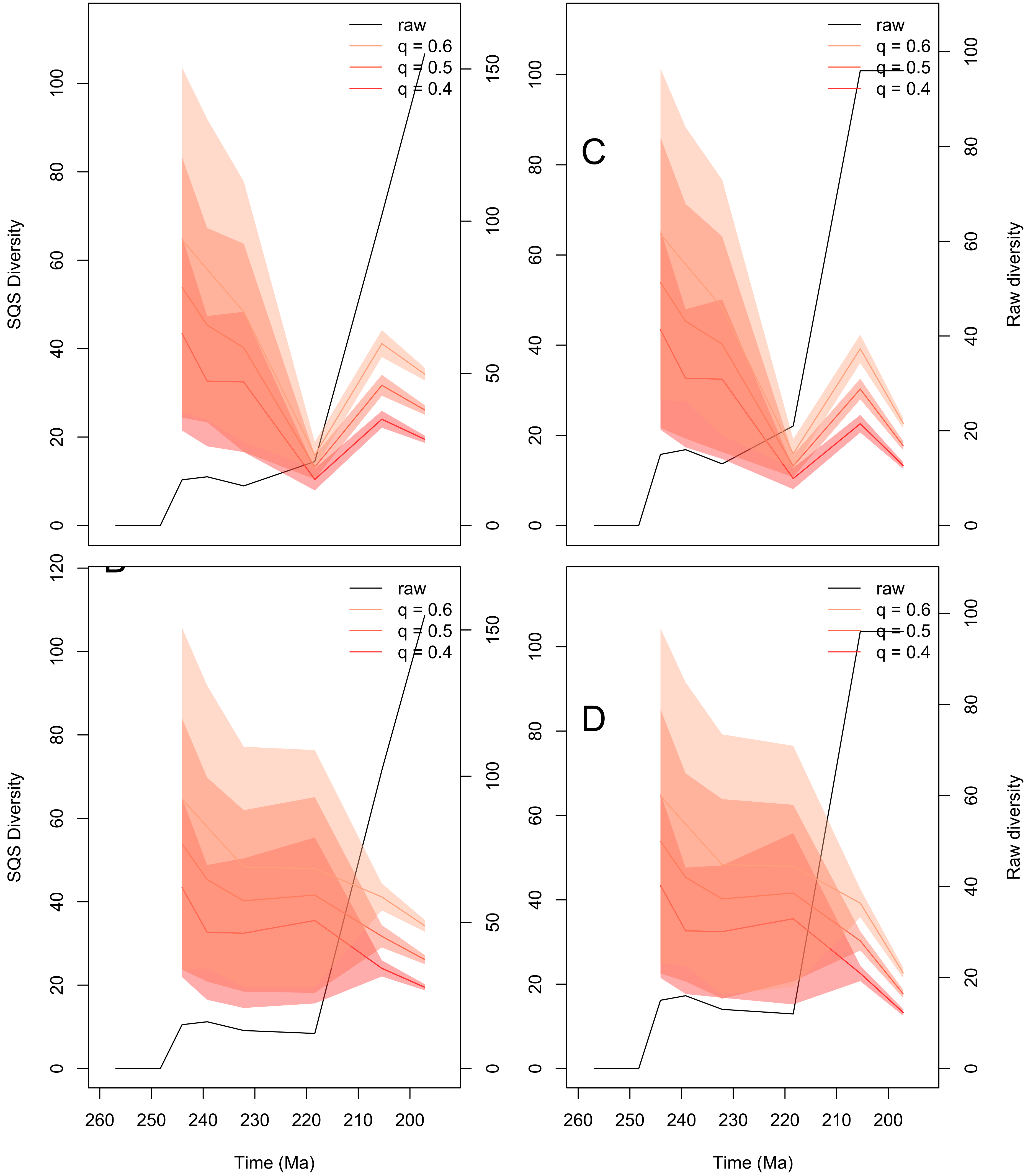

ementary Figure 14. Stage-wise sampling-standardised diversity for the South Panthalassic region under each data standardisation treat
(A) Unstandardised. (B) MST standardisation. (C) Longitude-latitude standardisation. (D) MST + longitude-latitude standardisation.

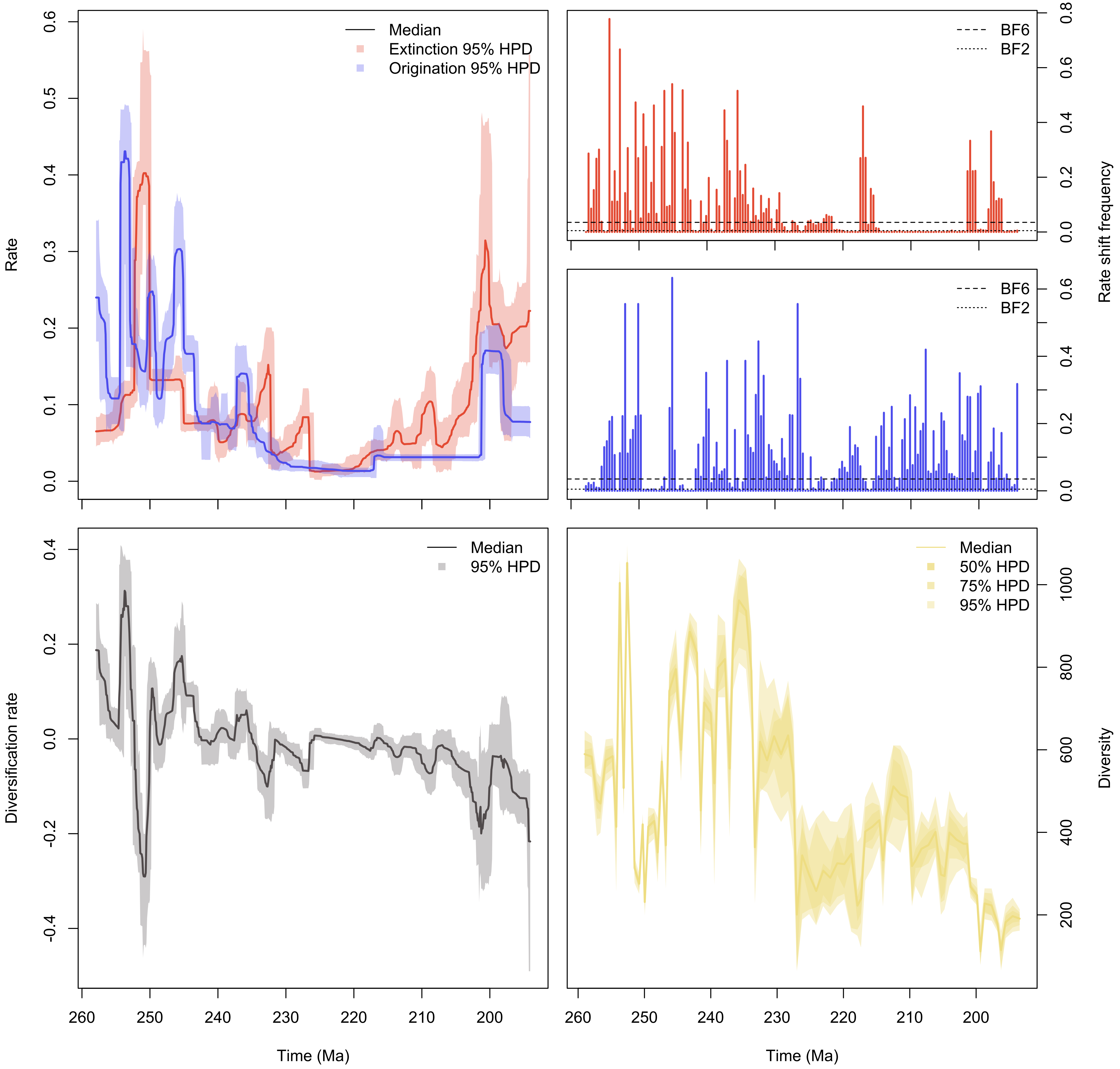

Supplementary Figure 15. Probabilistic origination, extinction and origination rates, and diversity for the Circumtethys region (unstandardised)

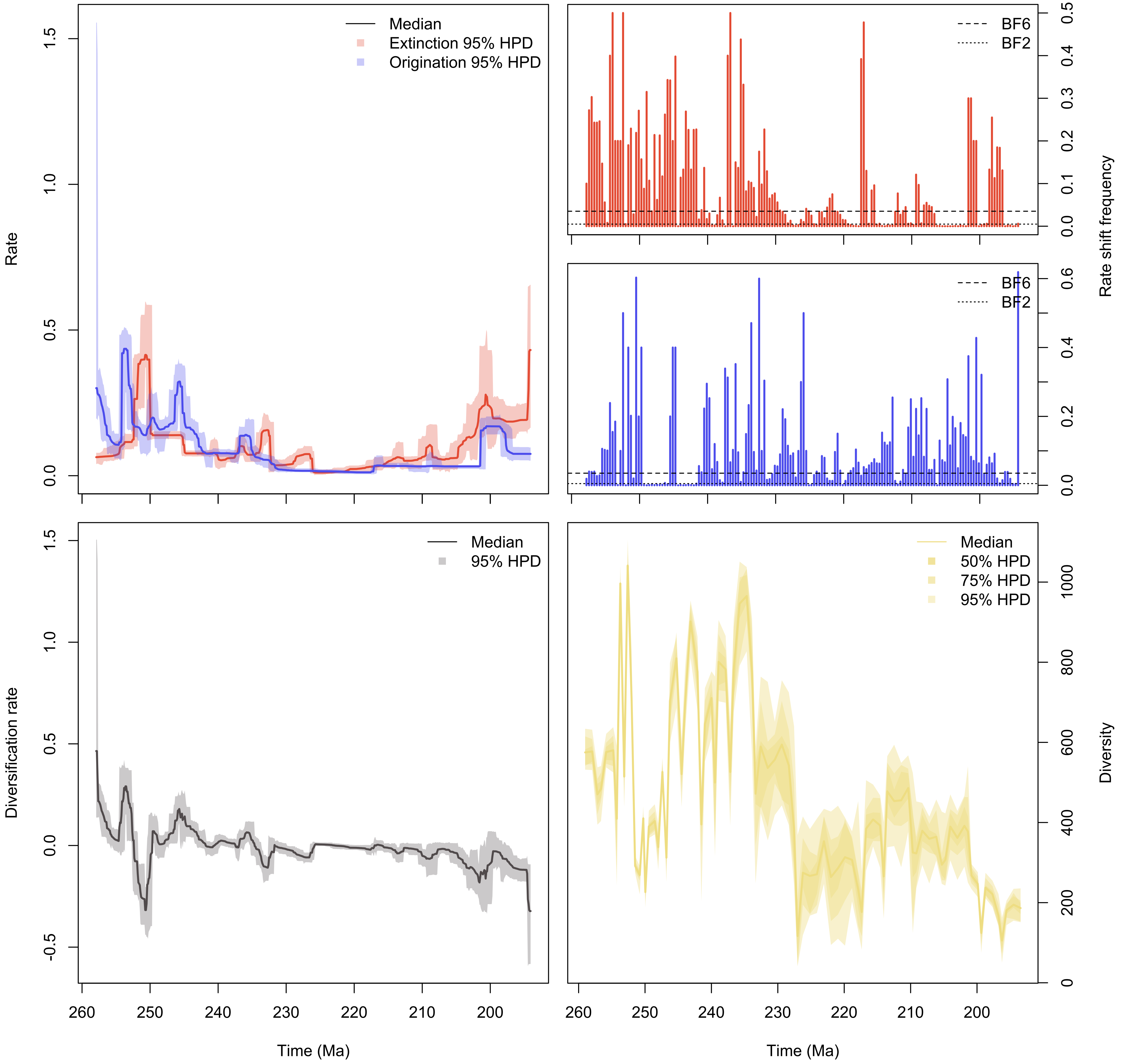

Supplementary Figure 16. Probabilistic origination, extinction and origination rates, and diversity for the Circumtethys region (MST standardised)

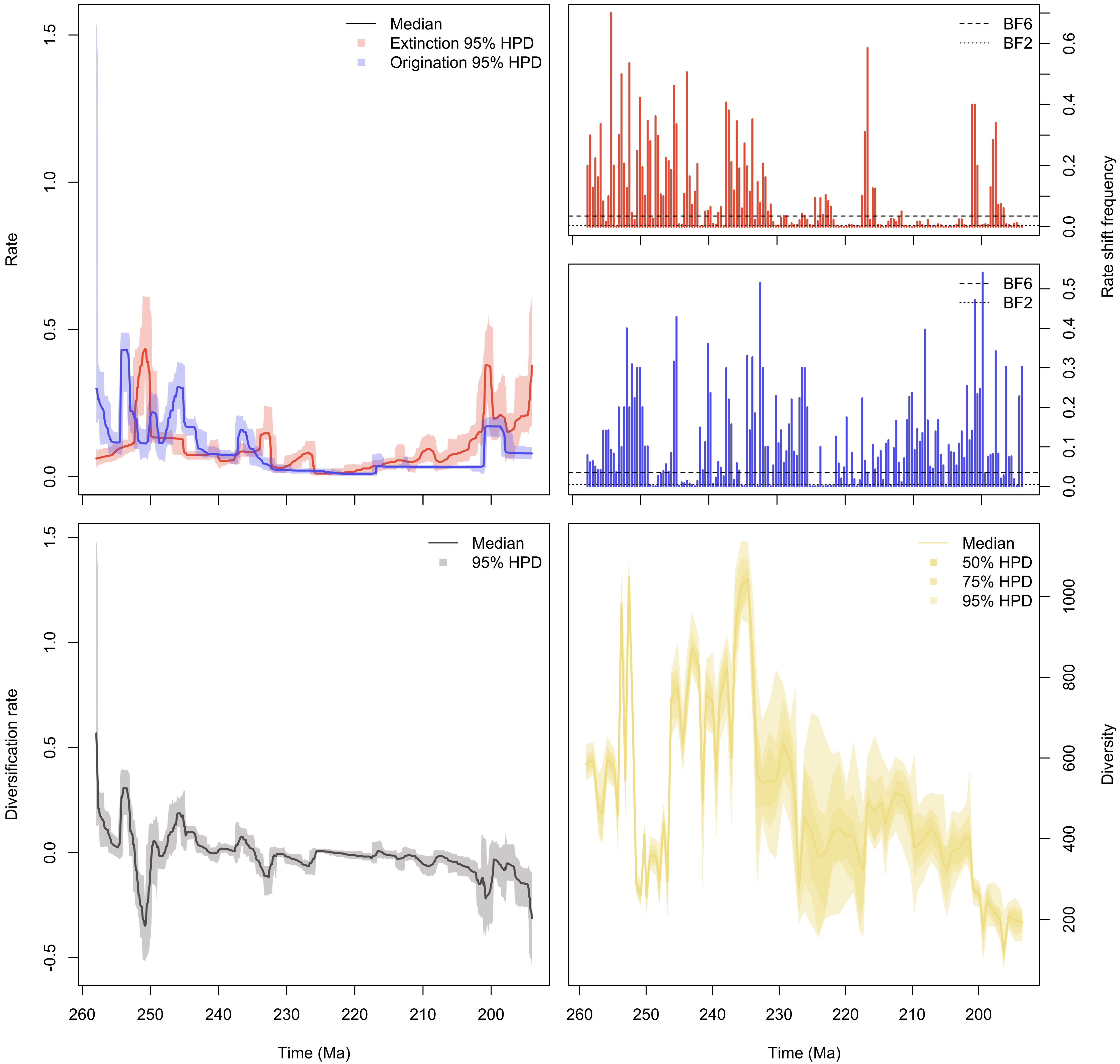

Supplementary Figure 17. Probabilistic origination, extinction and origination rates, and diversity for the Circumtethys region (MST + lng-lat standardised)

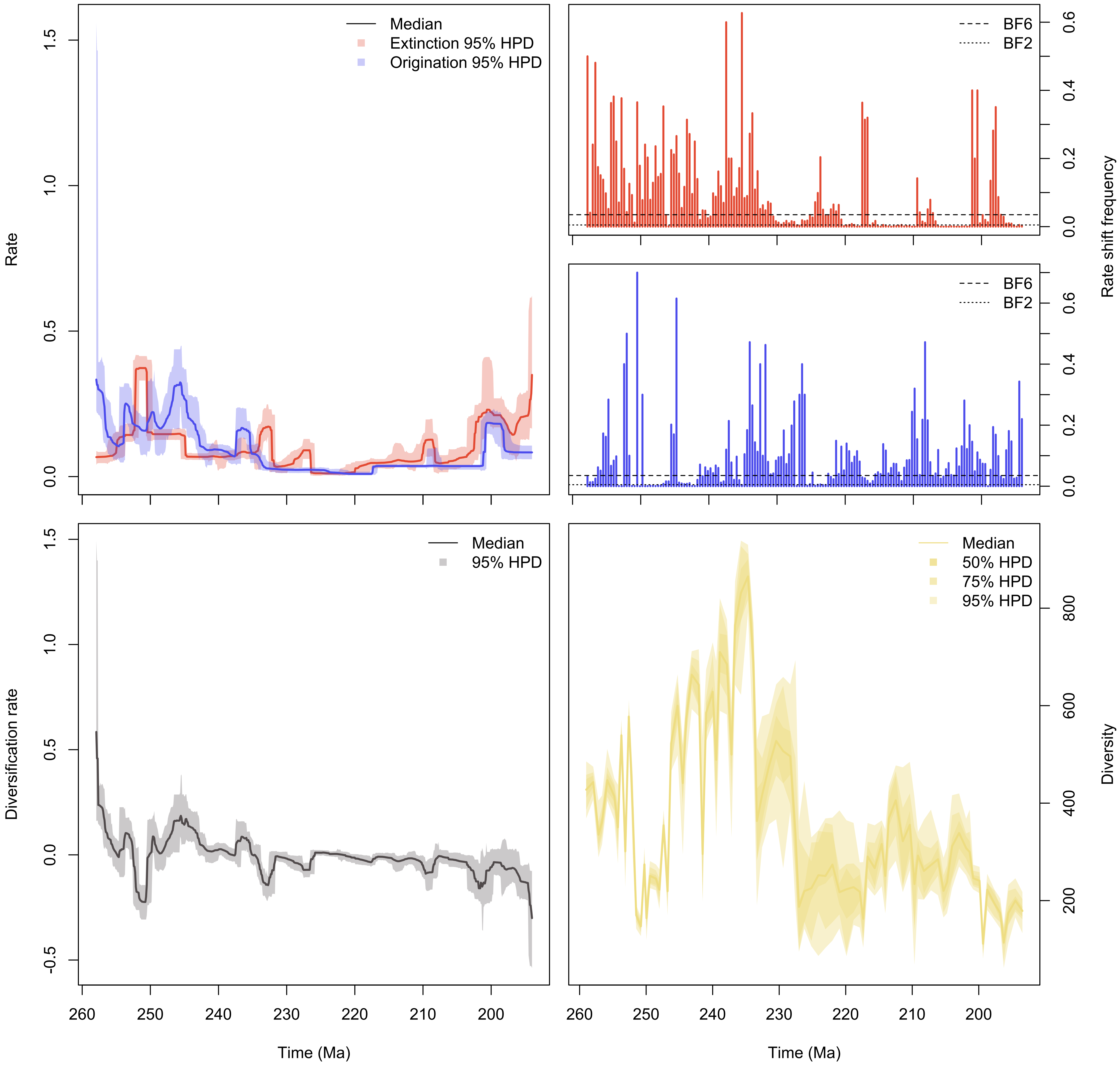

Supplementary Figure 18. Probabilistic origination, extinction and origination rates, and diversity for the West Circumtethys region (unstandardised)

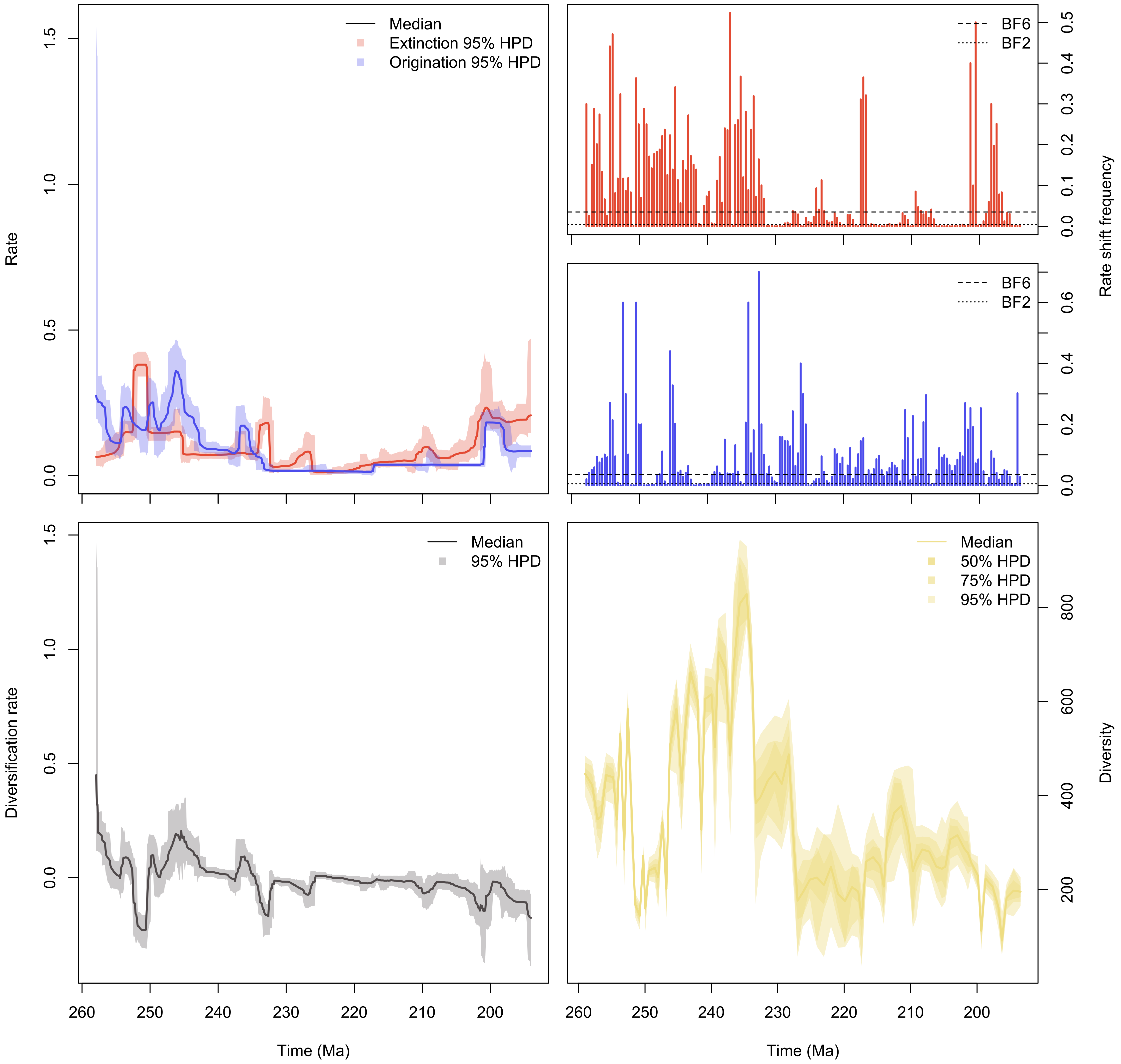

Supplementary Figure 19. Probabilistic origination, extinction and origination rates, and diversity for the West Circumtethys region (MST standardised)

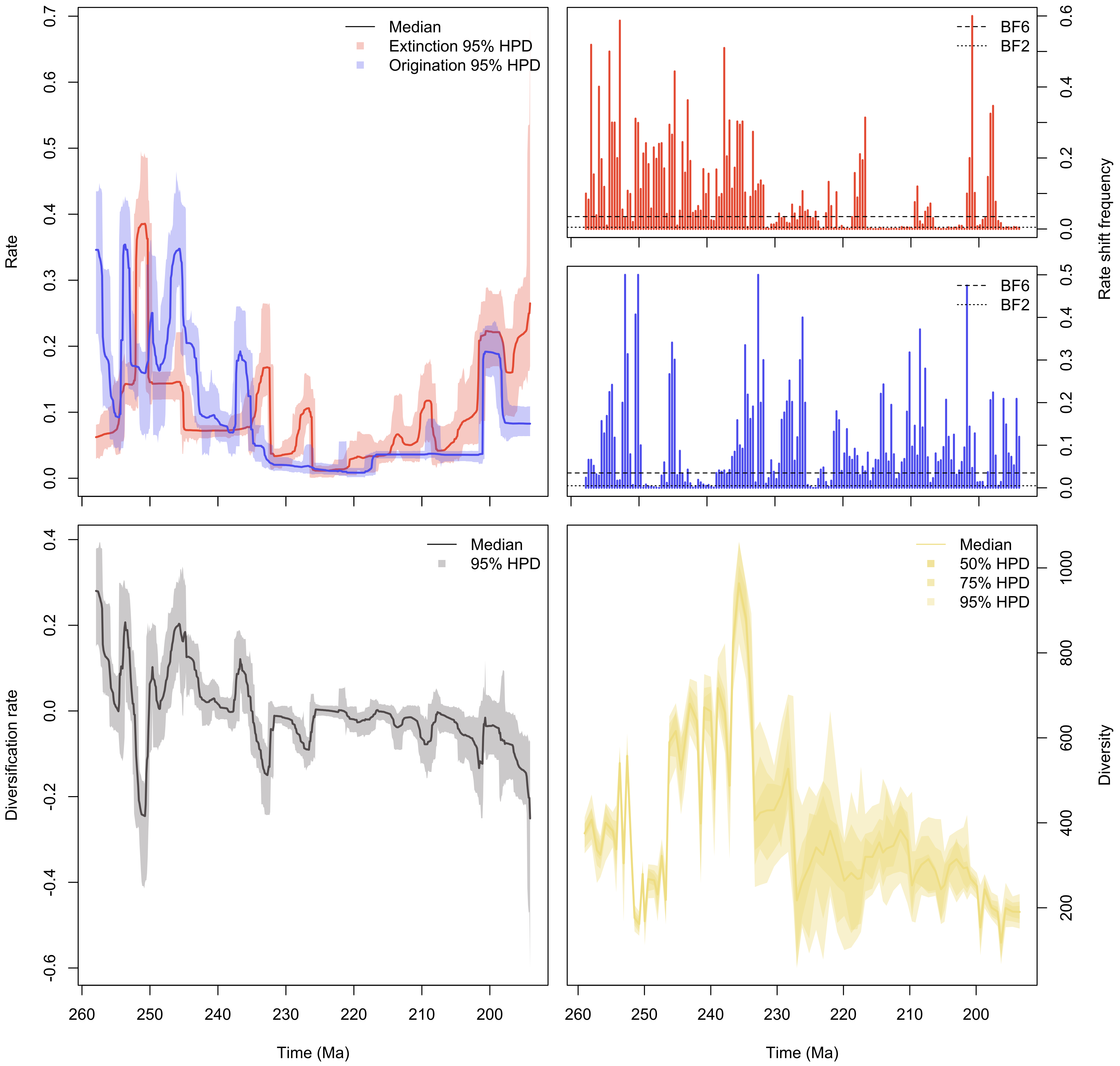

Supplementary Figure 20. Probabilistic origination, extinction and origination rates, and diversity for the West Circumtethys region (MST + lng-lat standardised)

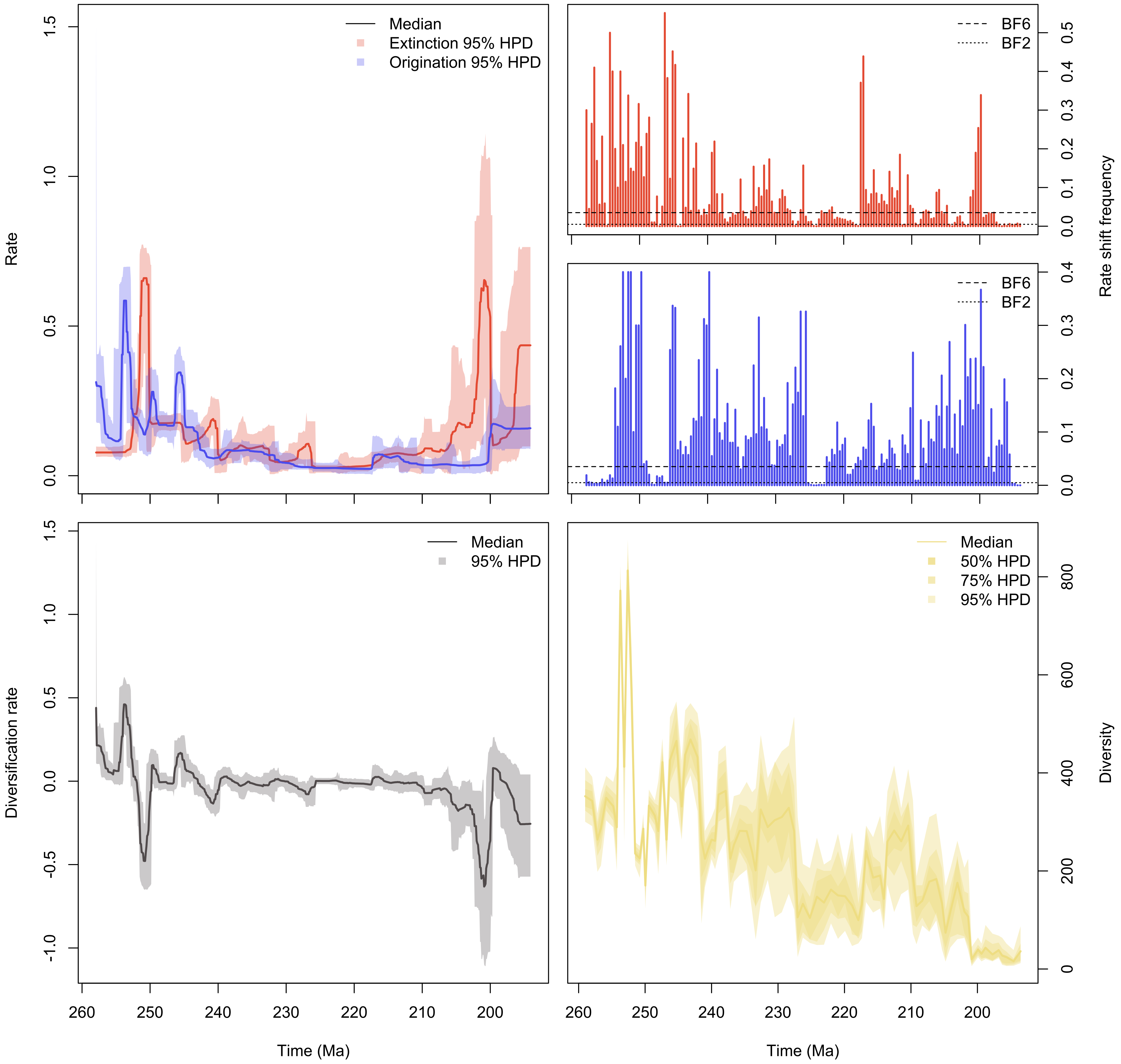

Supplementary Figure 21. Probabilistic origination, extinction and origination rates, and diversity for the East Circumtethys region (unstandardised)

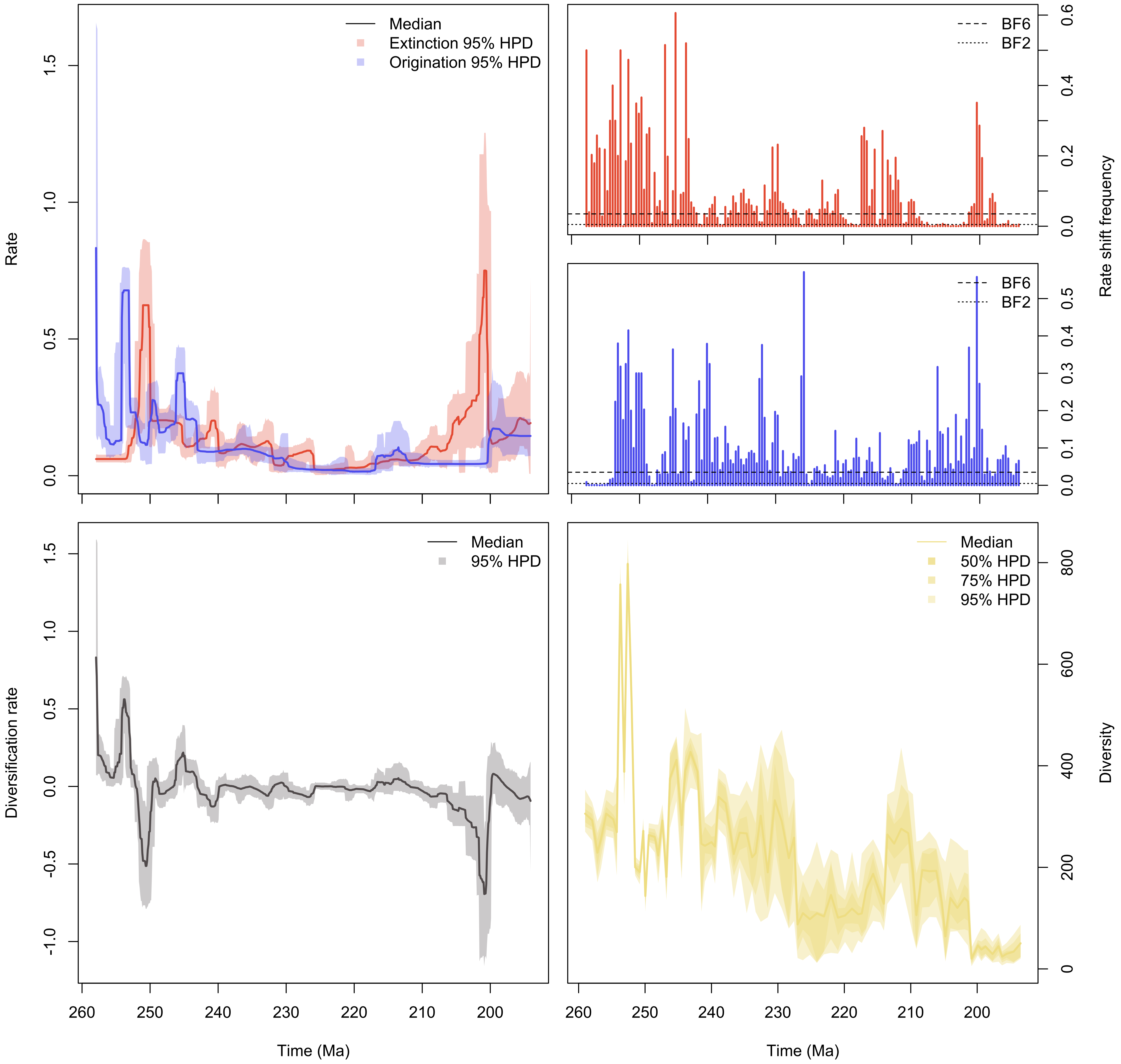

Supplementary Figure 22. Probabilistic origination, extinction and origination rates, and diversity for the East Circumtethys region (MST standardised)

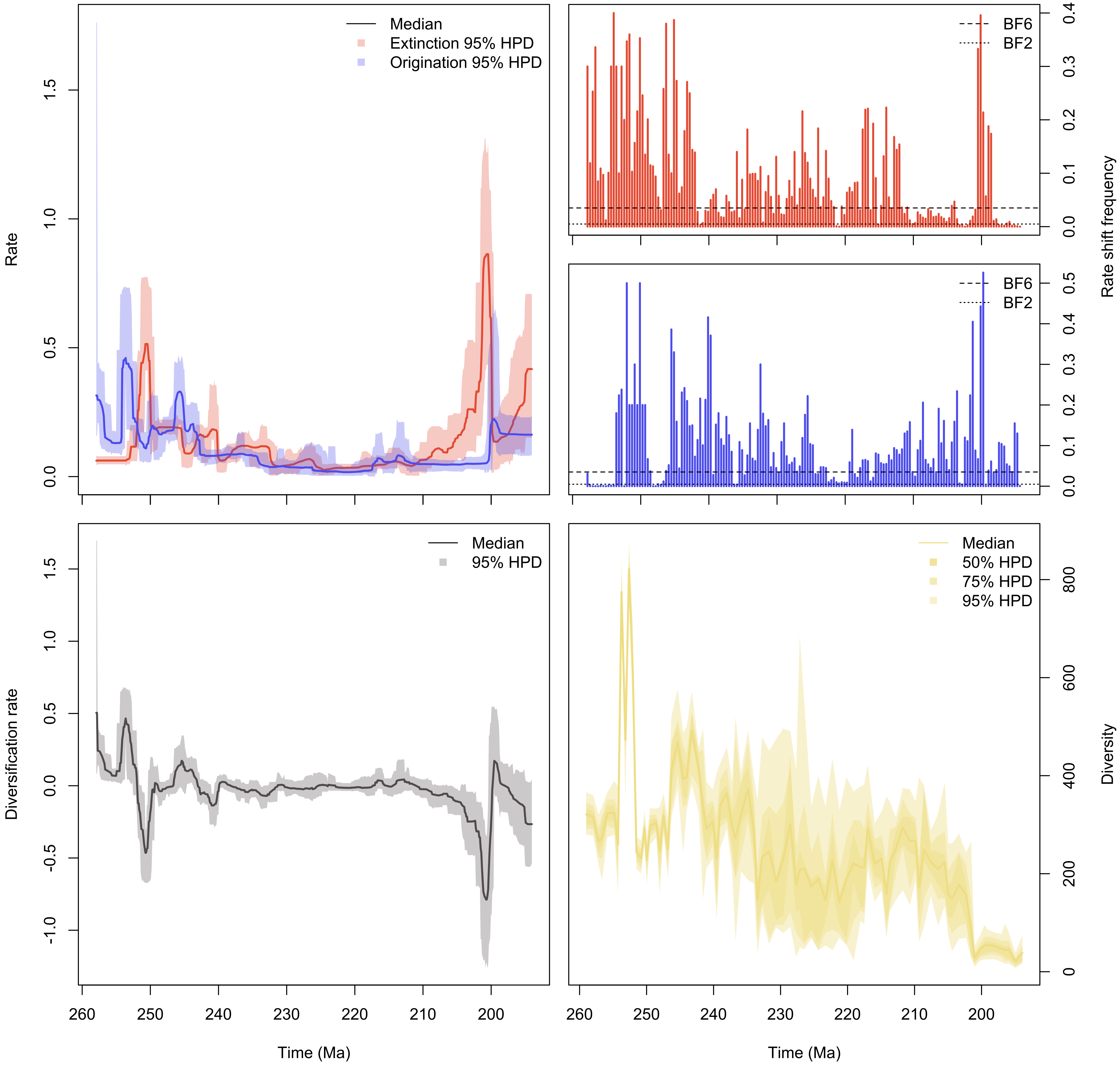

Supplementary Figure 23. Probabilistic origination, extinction and origination rates, and diversity for the East Circumtethys region (MST + lng-lat standardised)

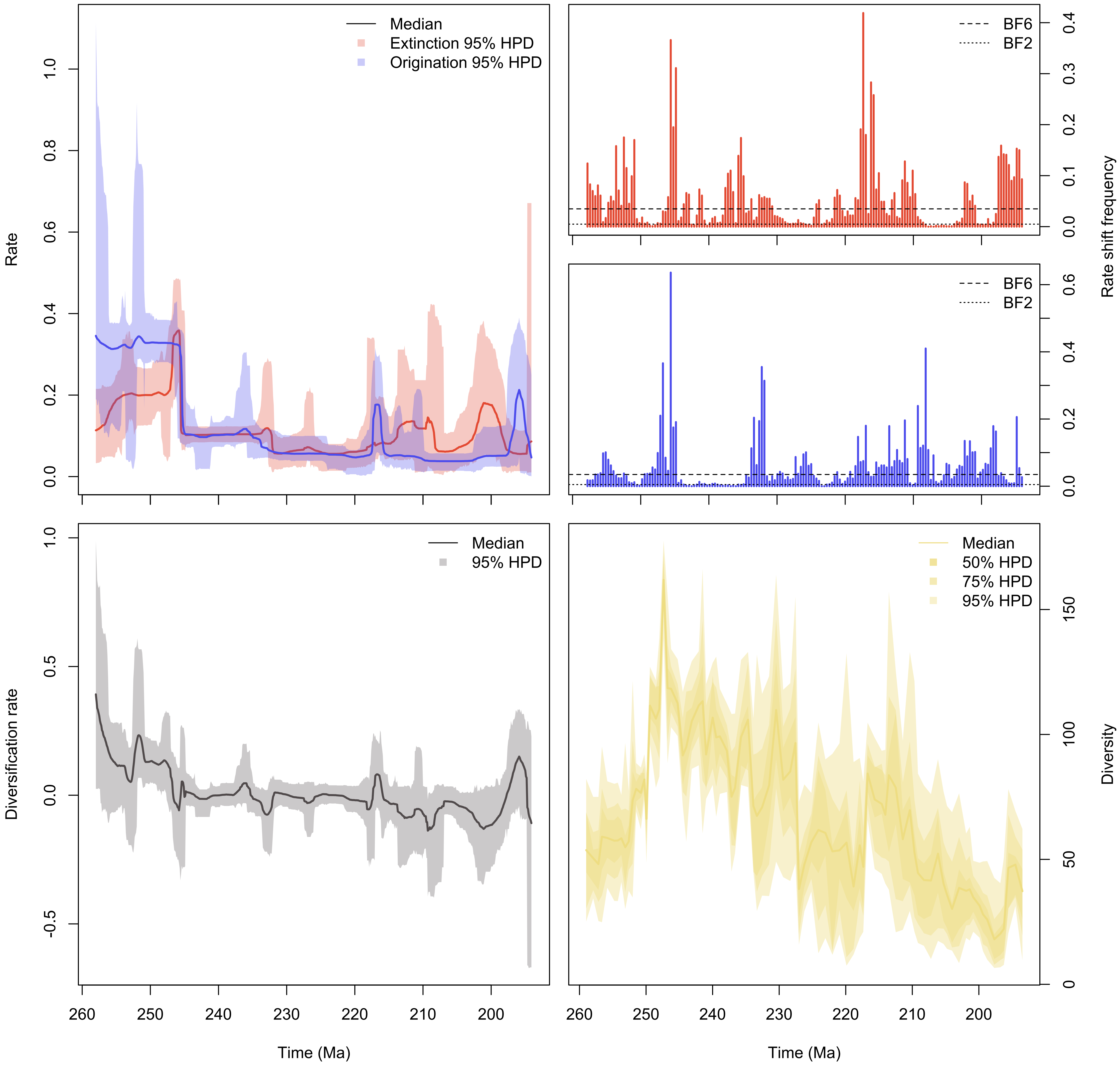

Supplementary Figure 24. Probabilistic origination, extinction and origination rates, and diversity for the Boreal region (unstandardised)

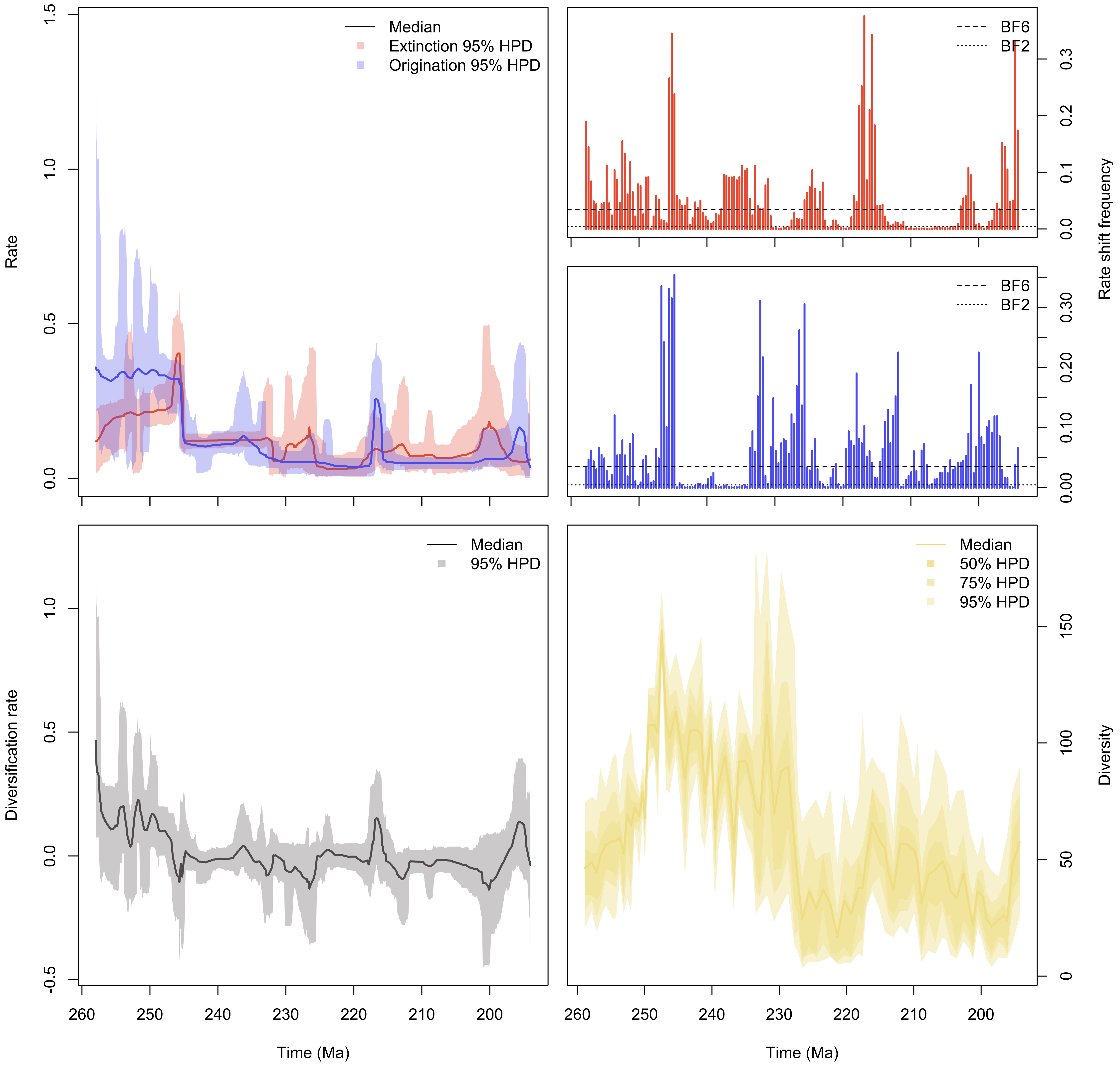

Supplementary Figure 25. Probabilistic origination, extinction and origination rates, and diversity for the Boreal region (MST standardised)

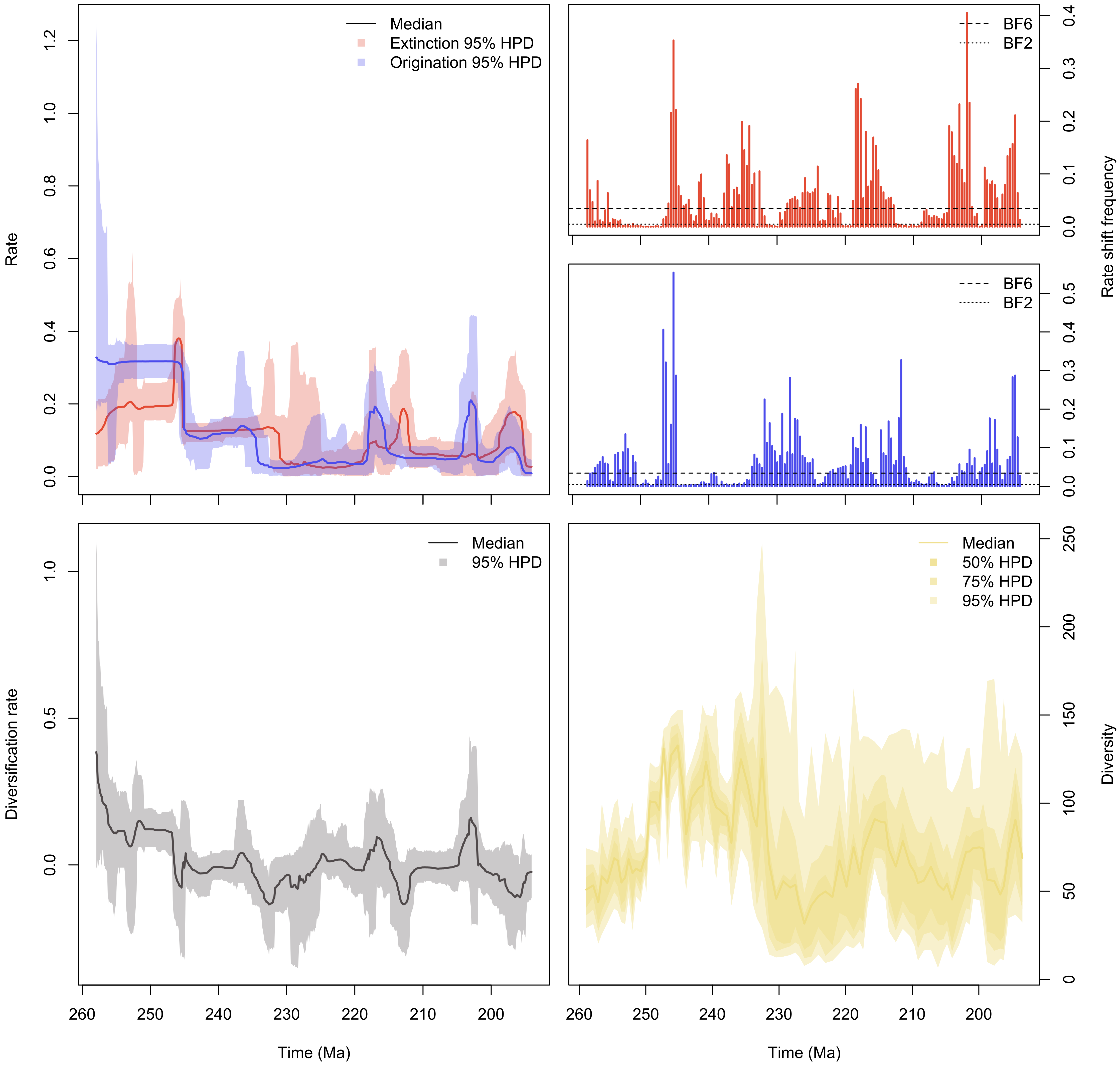

Supplementary Figure 26. Probabilistic origination, extinction and origination rates, and diversity for the Boreal region (MST + lng-lat standardised)

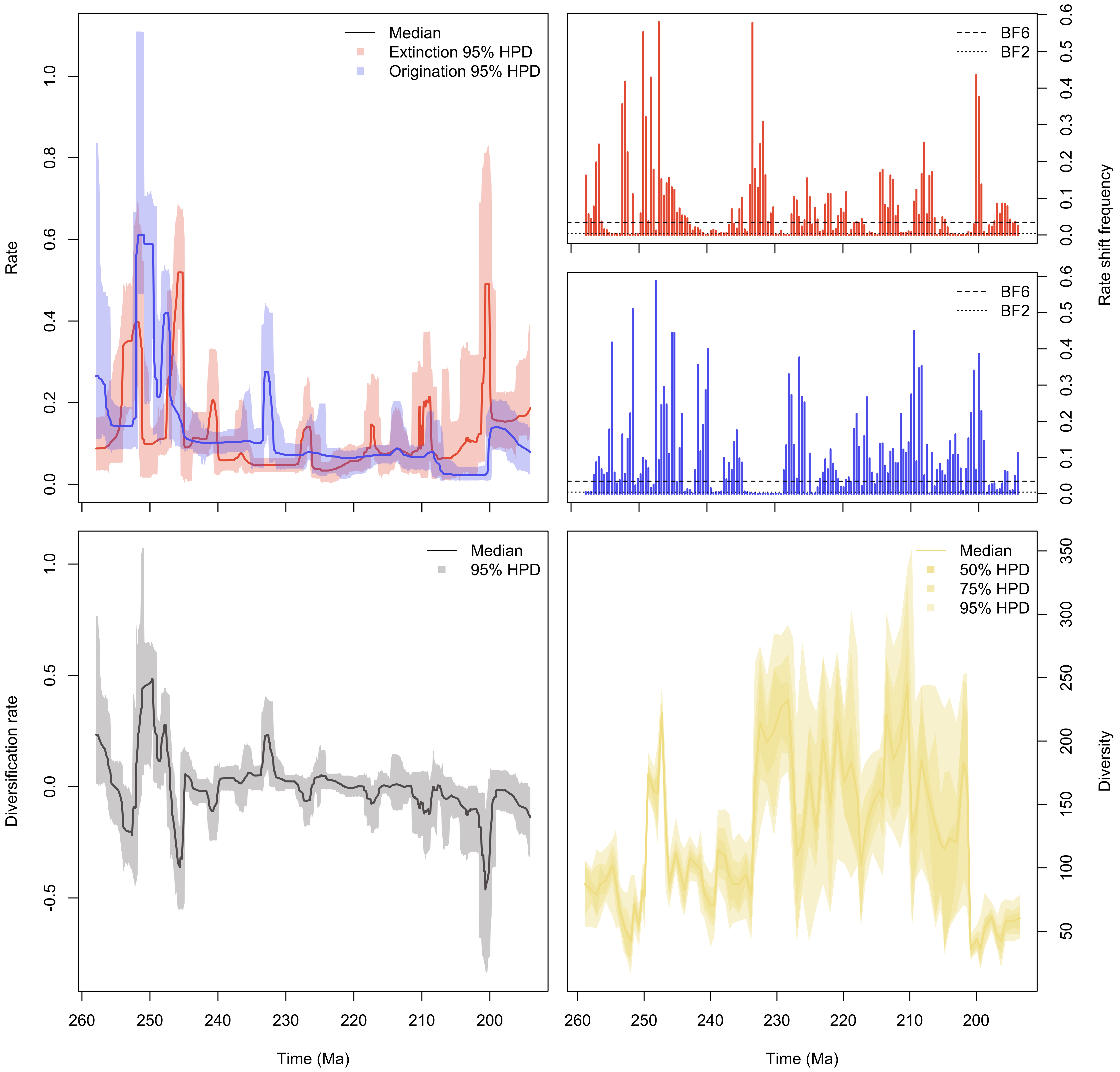

Supplementary Figure 27. Probabilistic origination, extinction and origination rates, and diversity for the North Panthalassic region (unstandardised)

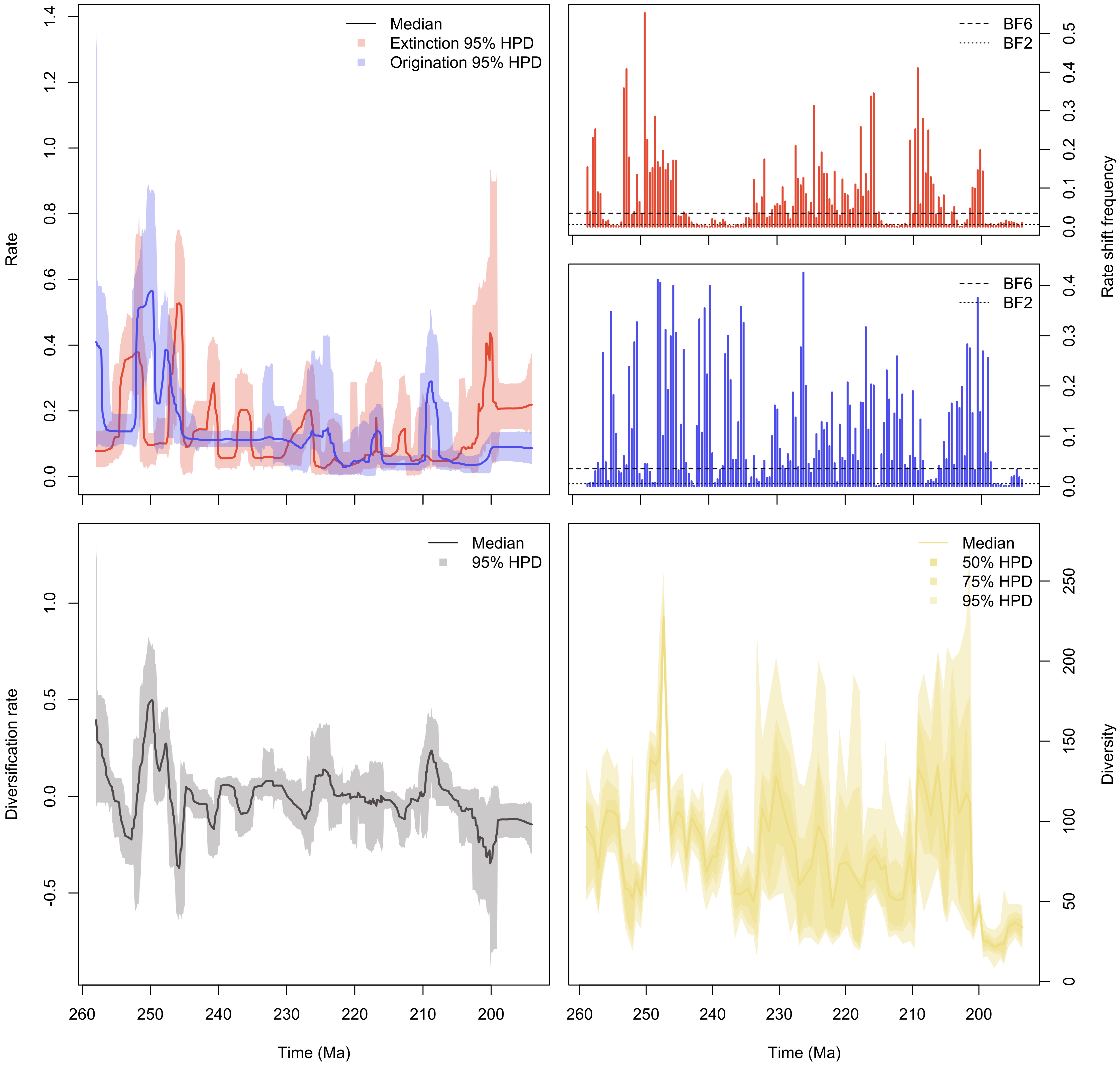

Supplementary Figure 28. Probabilistic origination, extinction and origination rates, and diversity for the North Panthalassic region (MST standardised)

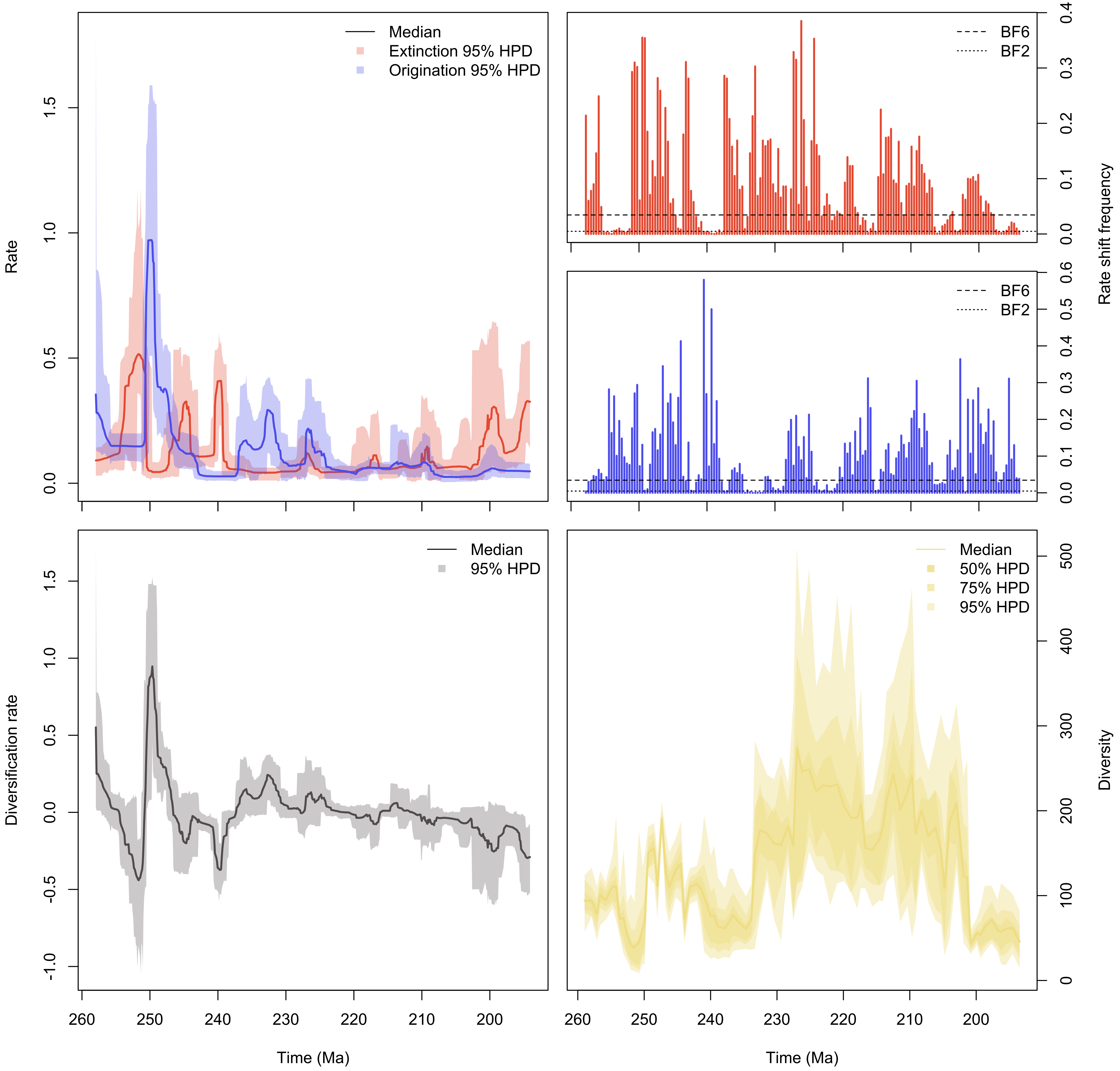

Supplementary Figure 29. Probabilistic origination, extinction and origination rates, and diversity for the North Panthalassic region (MST + lng-lat standardised)

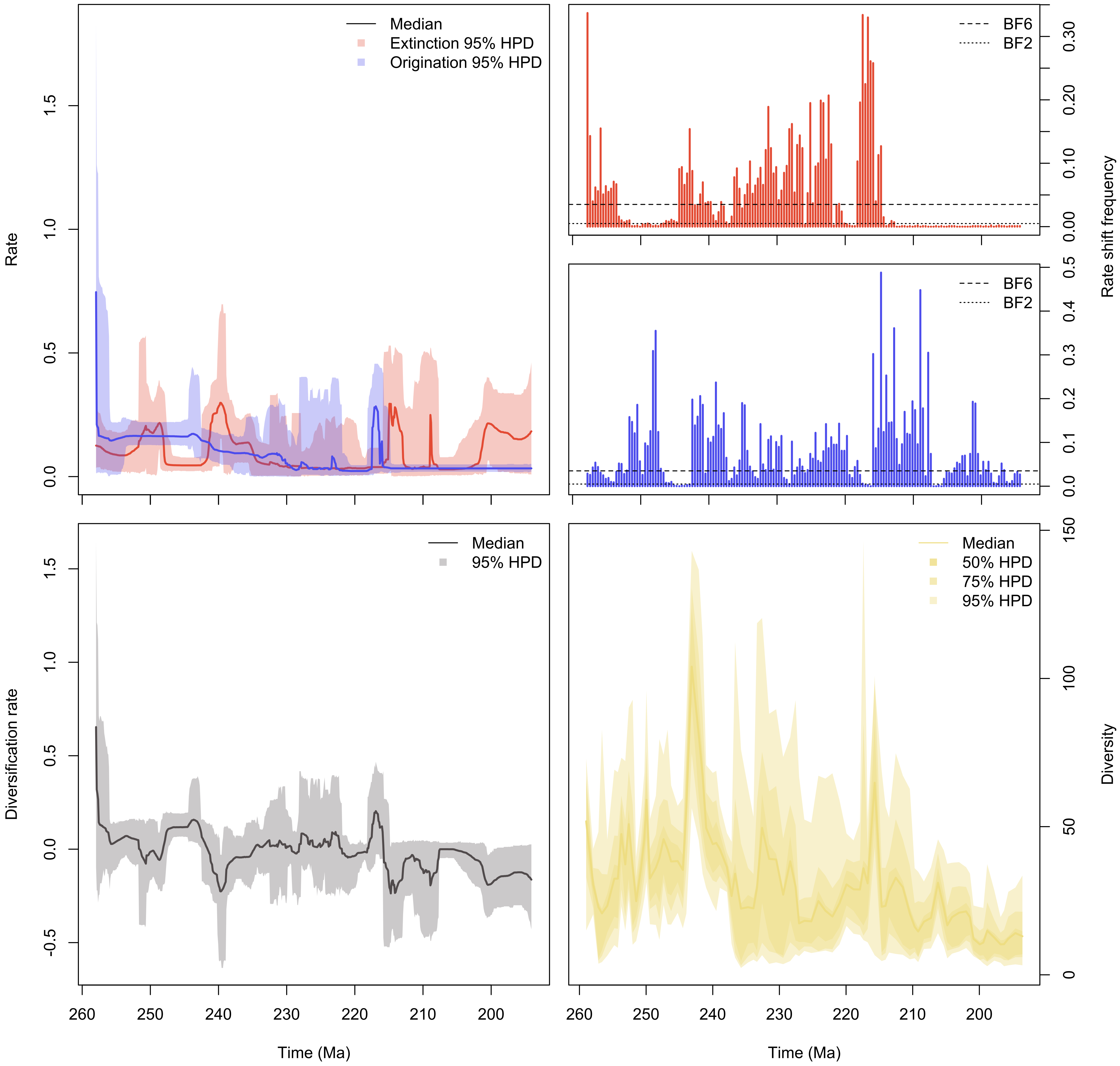

Supplementary Figure 30. Probabilistic origination, extinction and origination rates, and diversity for the Tangaroan region (unstandardised)

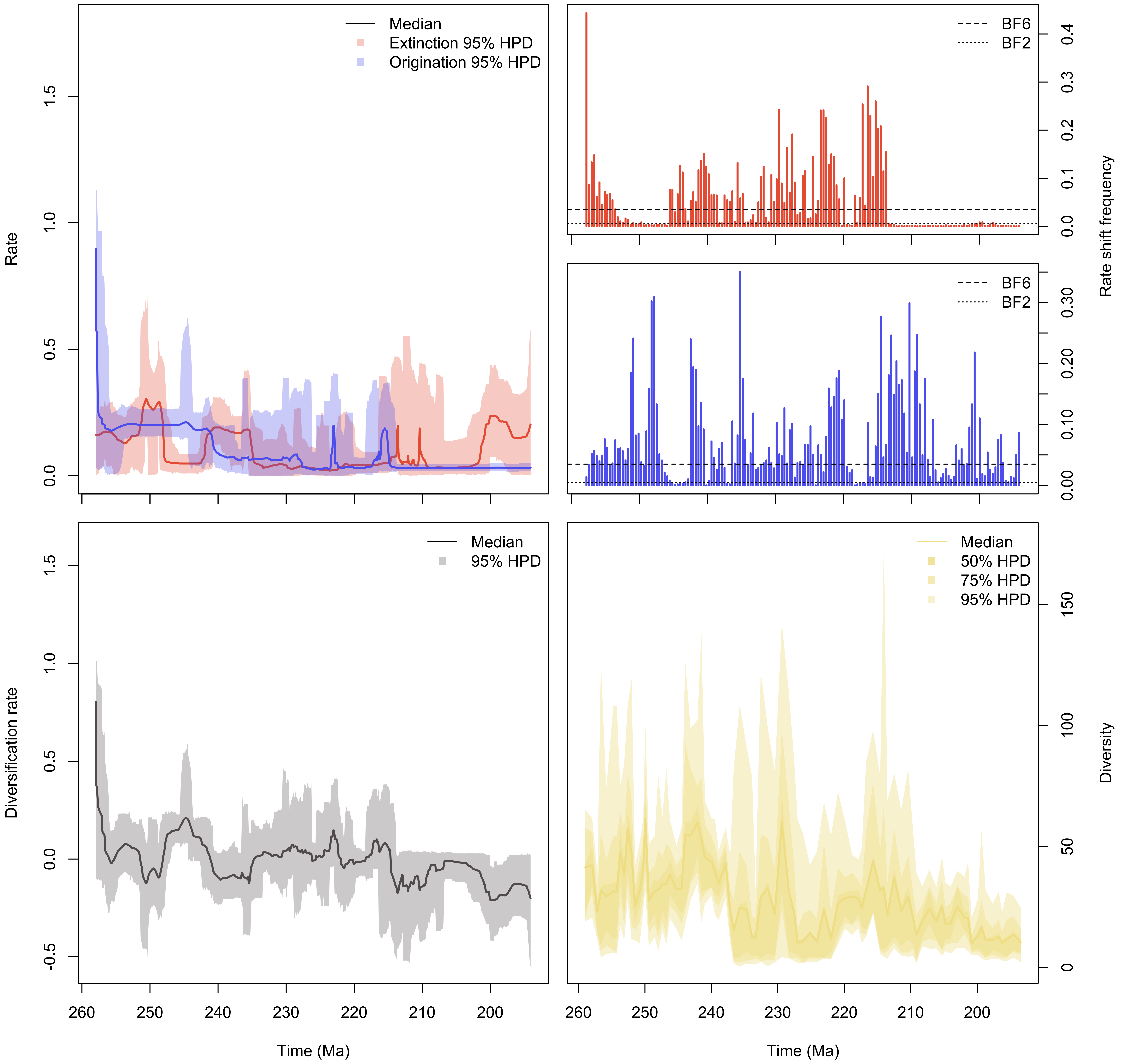

Supplementary Figure 31. Probabilistic origination, extinction and origination rates, and diversity for the Tangaroan region (MST standardised)

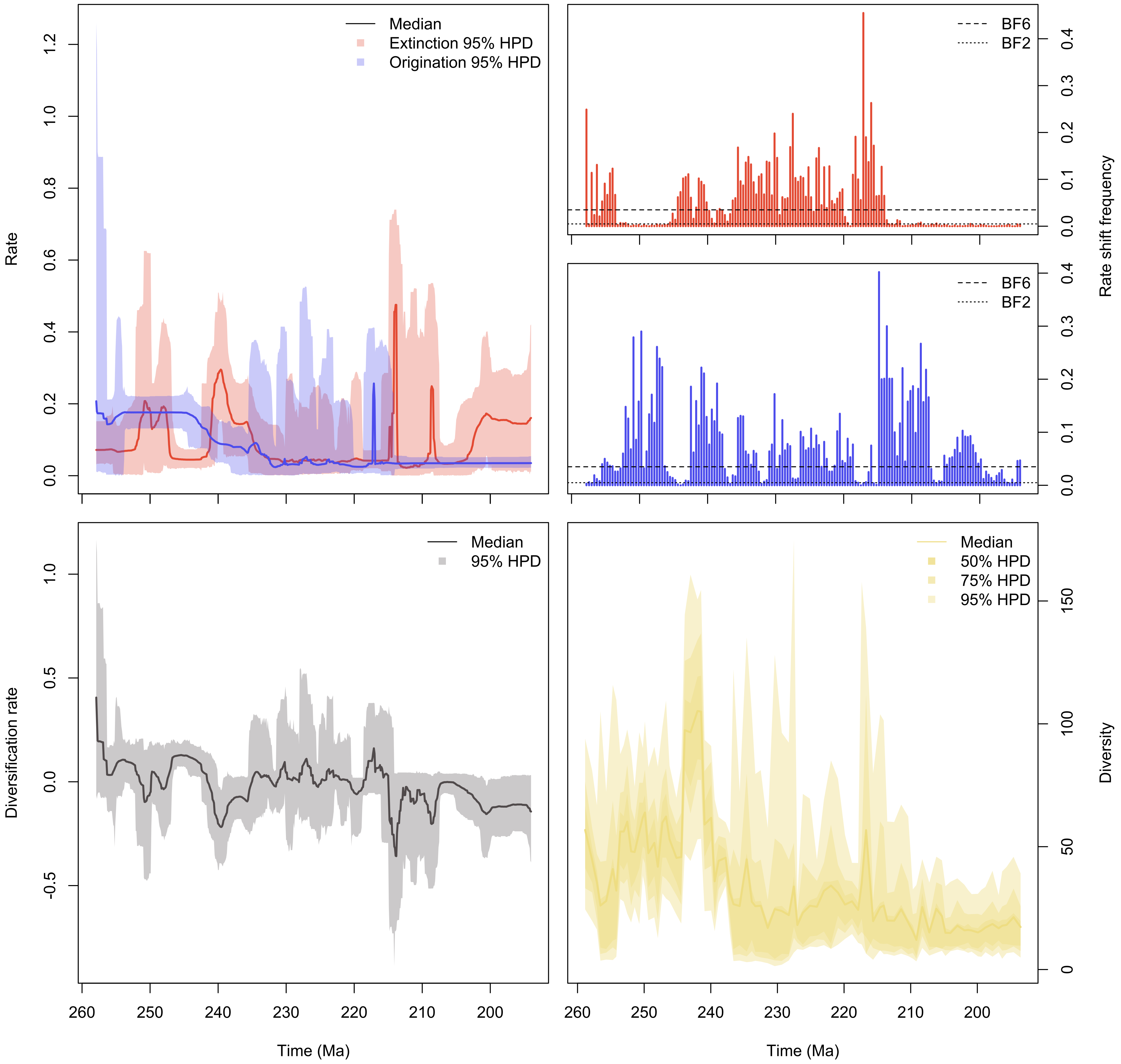

Supplementary Figure 32. Probabilistic origination, extinction and origination rates, and diversity for the Tangaroan region (MST + lng-lat standardised)

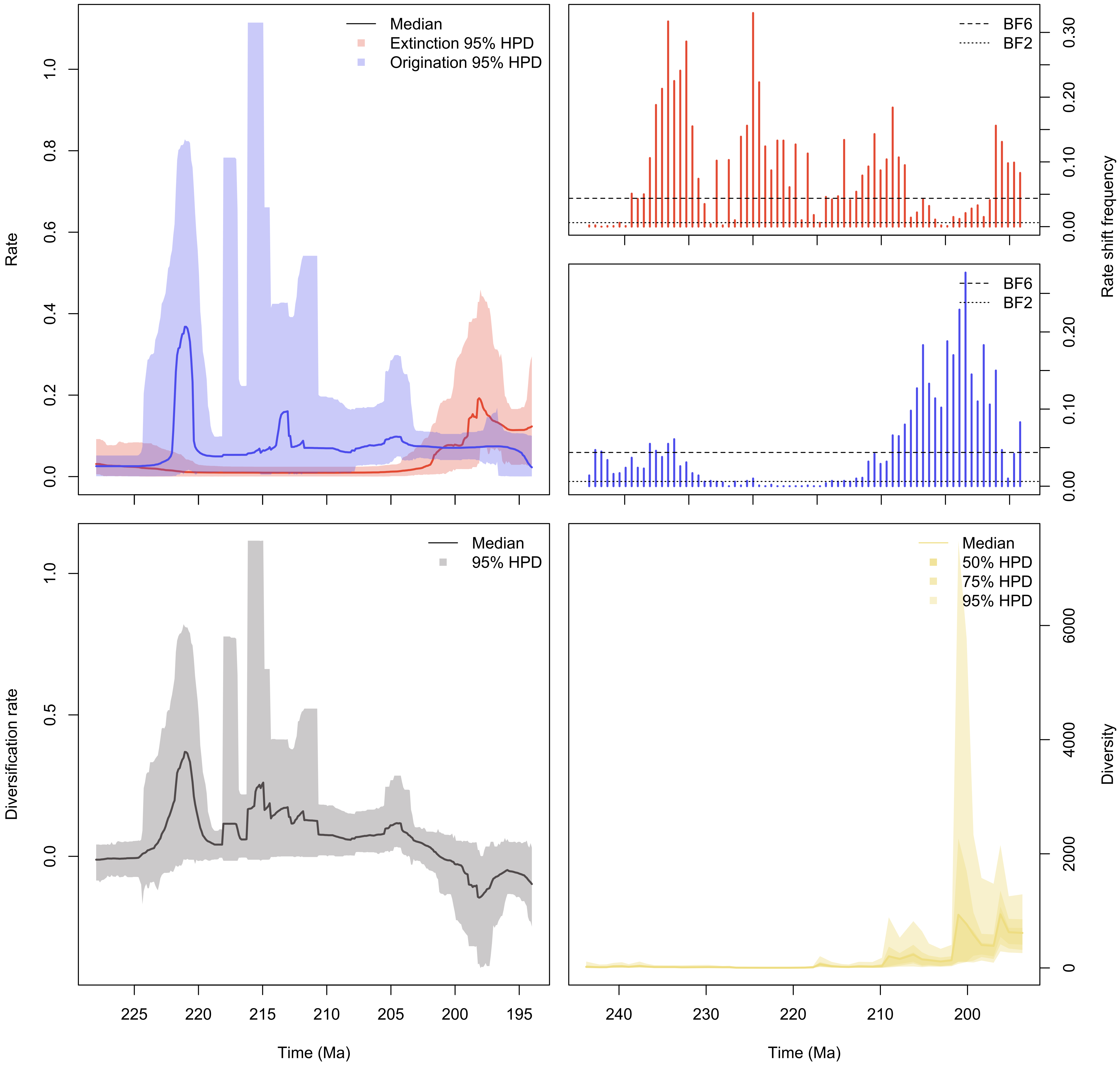

Supplementary Figure 33. Probabilistic origination, extinction and origination rates, and diversity for the South Panthalassic region (unstandardised)

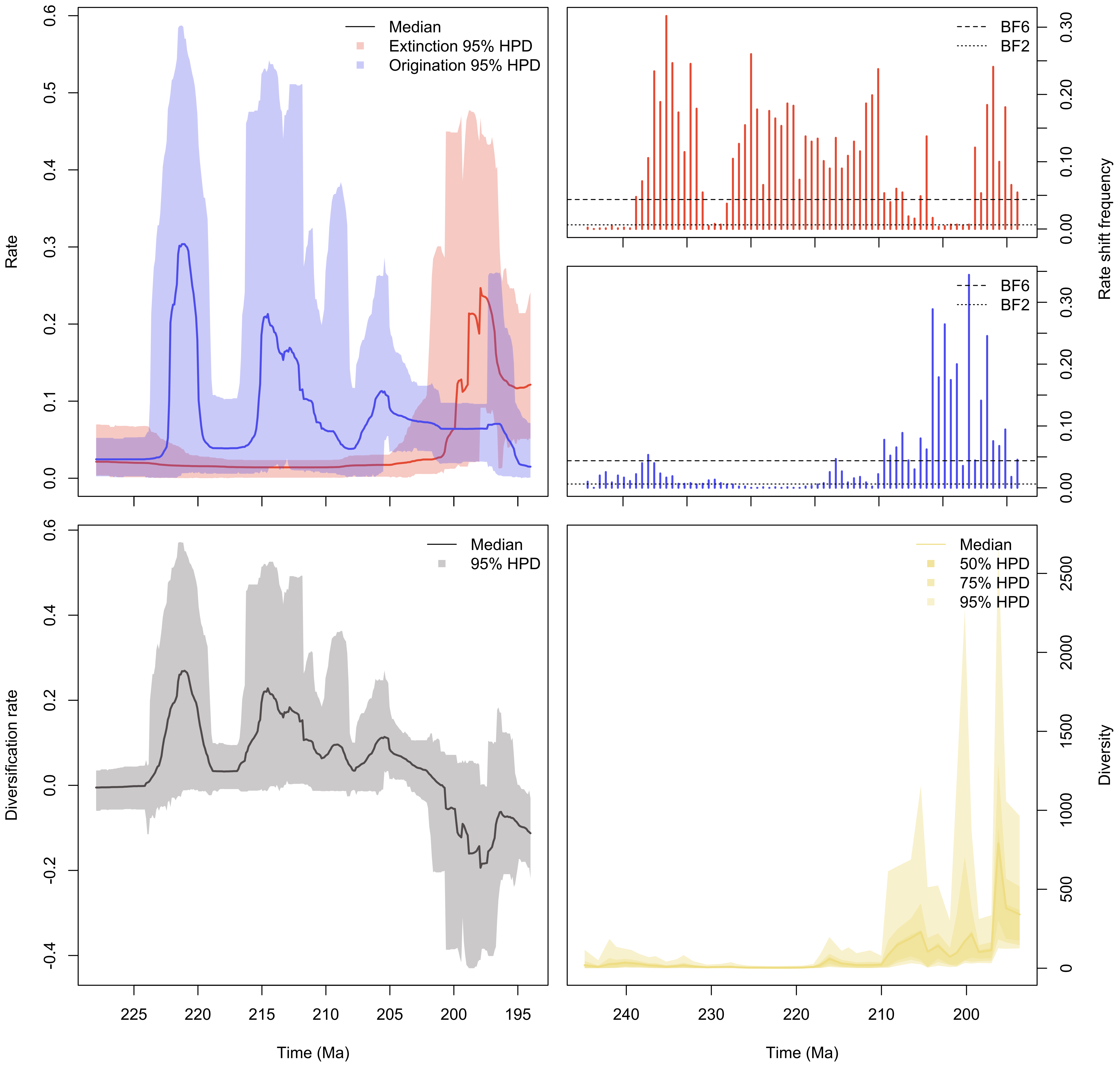

Supplementary Figure 34. Probabilistic origination, extinction and origination rates, and diversity for the South Panthalassic region (MST standardised)

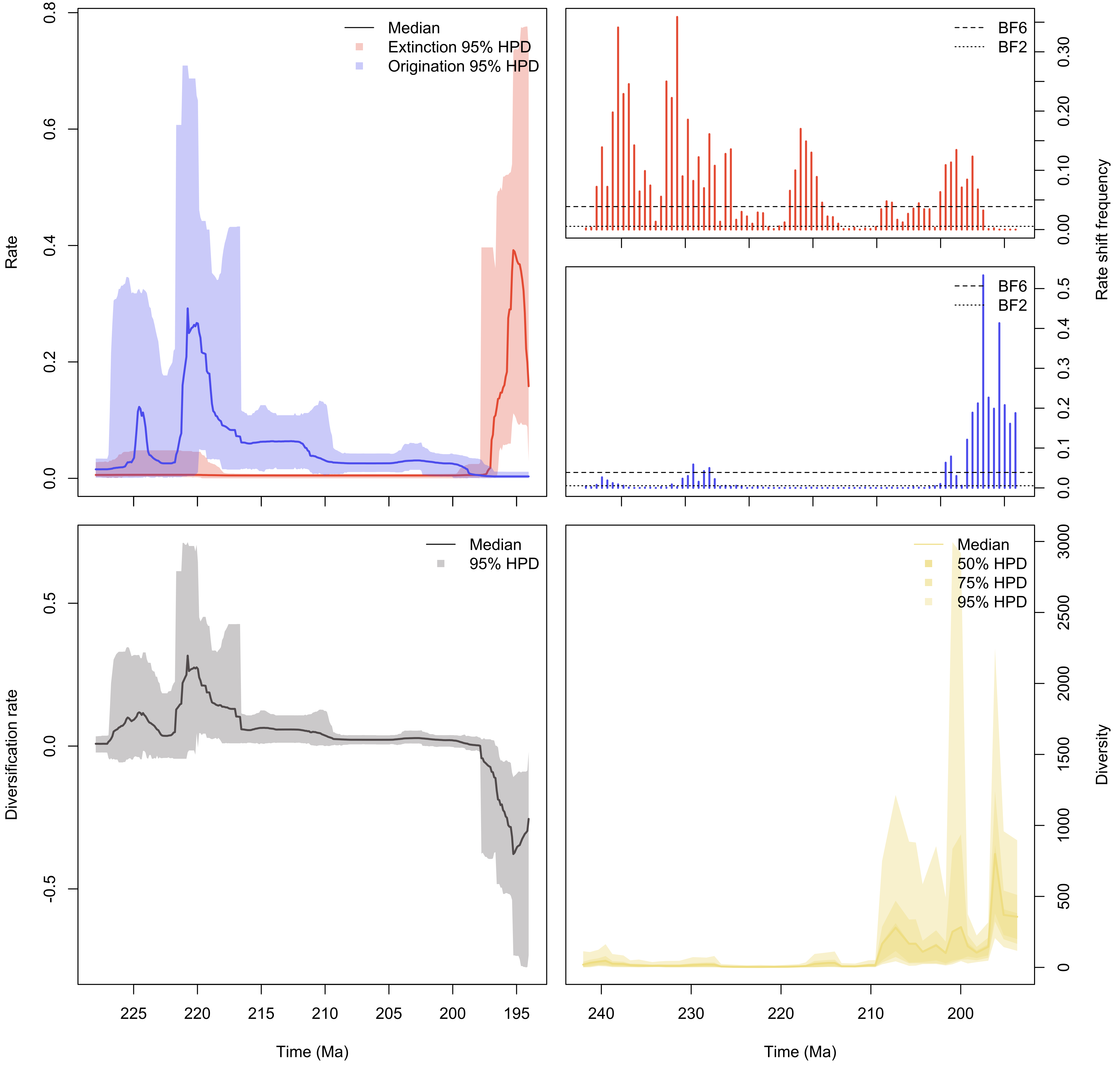

Supplementary Figure 35. Probabilistic origination, extinction and origination rates, and diversity for the South Panthalassic region (MST + lng-lat standardised)

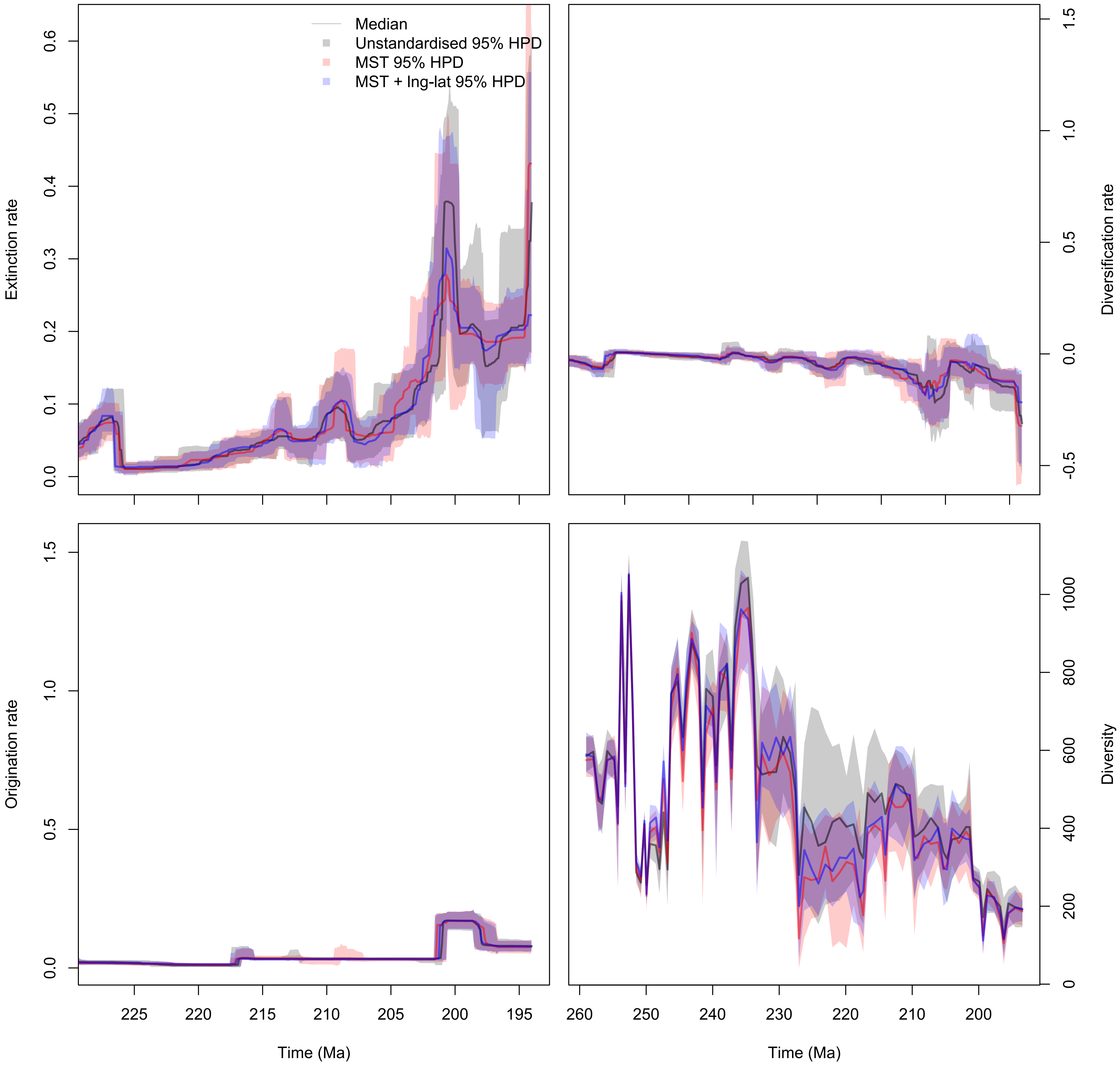

Supplementary Figure 36. Comparison of probabilistic origination, extinction and origination rates, and diversity under each data standardisation treatment for the Circumtethys region

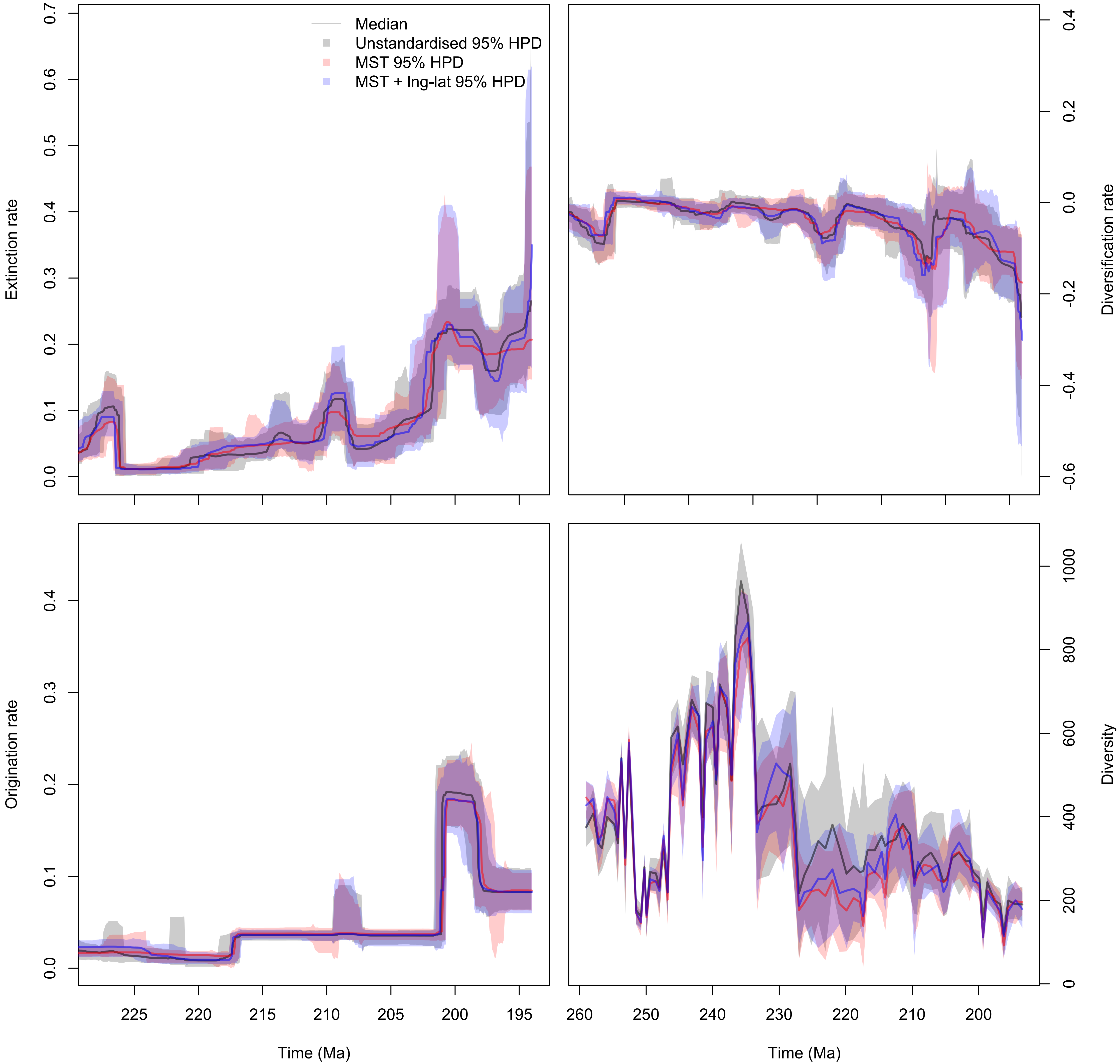

Supplementary Figure 37. Comparison of probabilistic origination, extinction and origination rates, and diversity under each data standardisation treatment for the West Circumtethys region

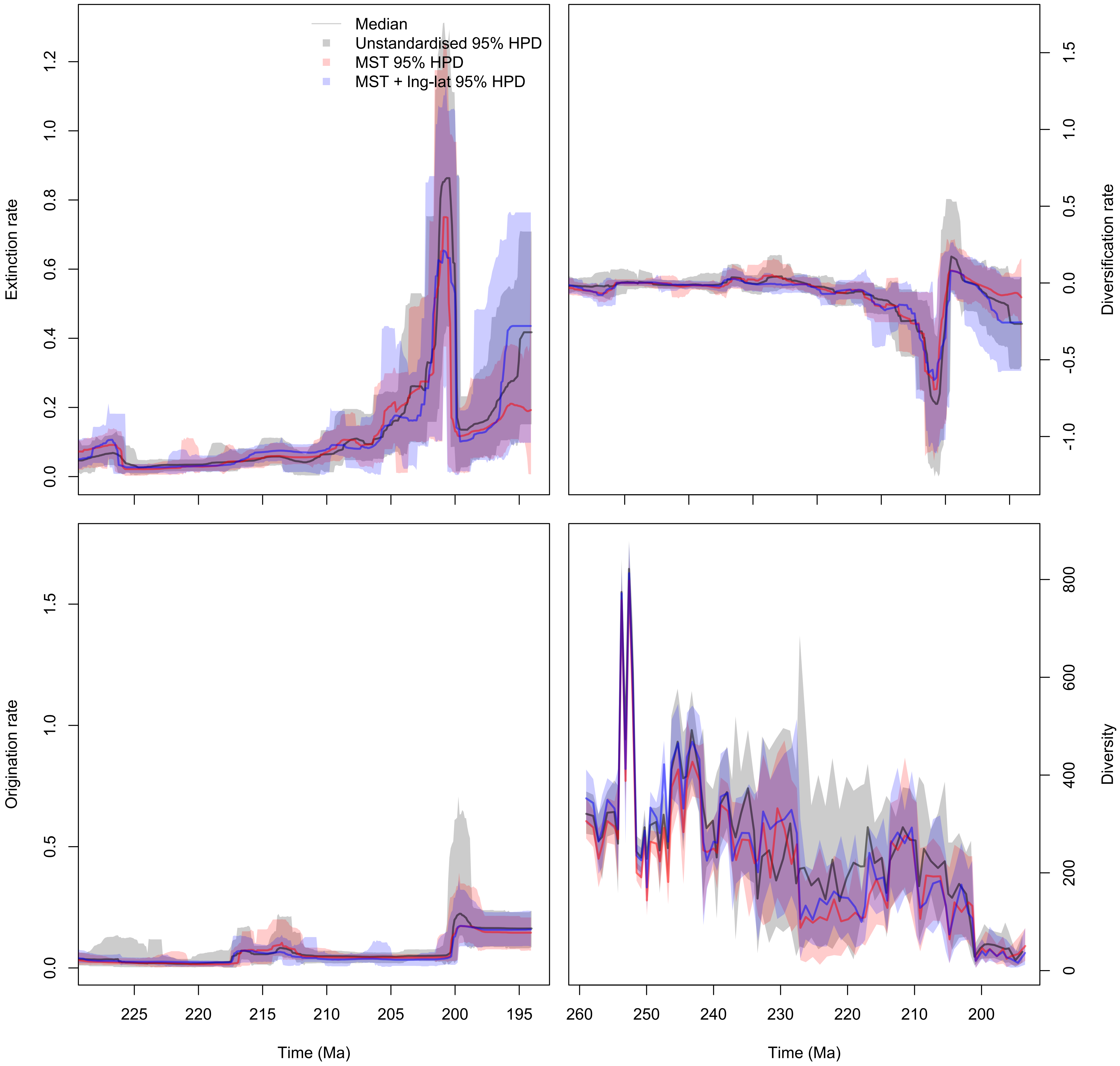

Supplementary Figure 38. Comparison of probabilistic origination, extinction and origination rates, and diversity under each data standardisation treatment for the East Circumtethys region

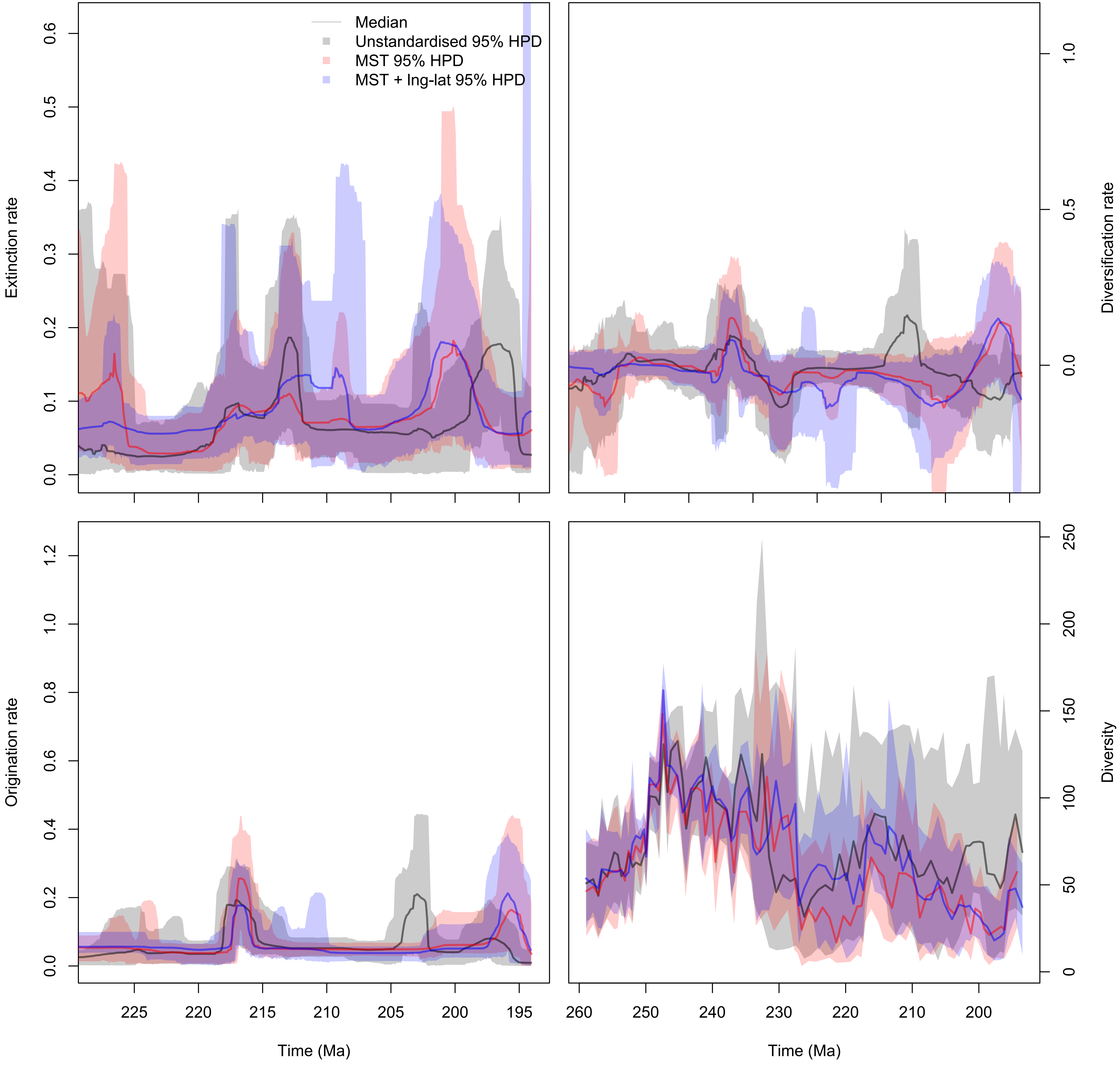

Supplementary Figure 39. Comparison of probabilistic origination, extinction and origination rates, and diversity under each data standardisation treatment for the Boreal region

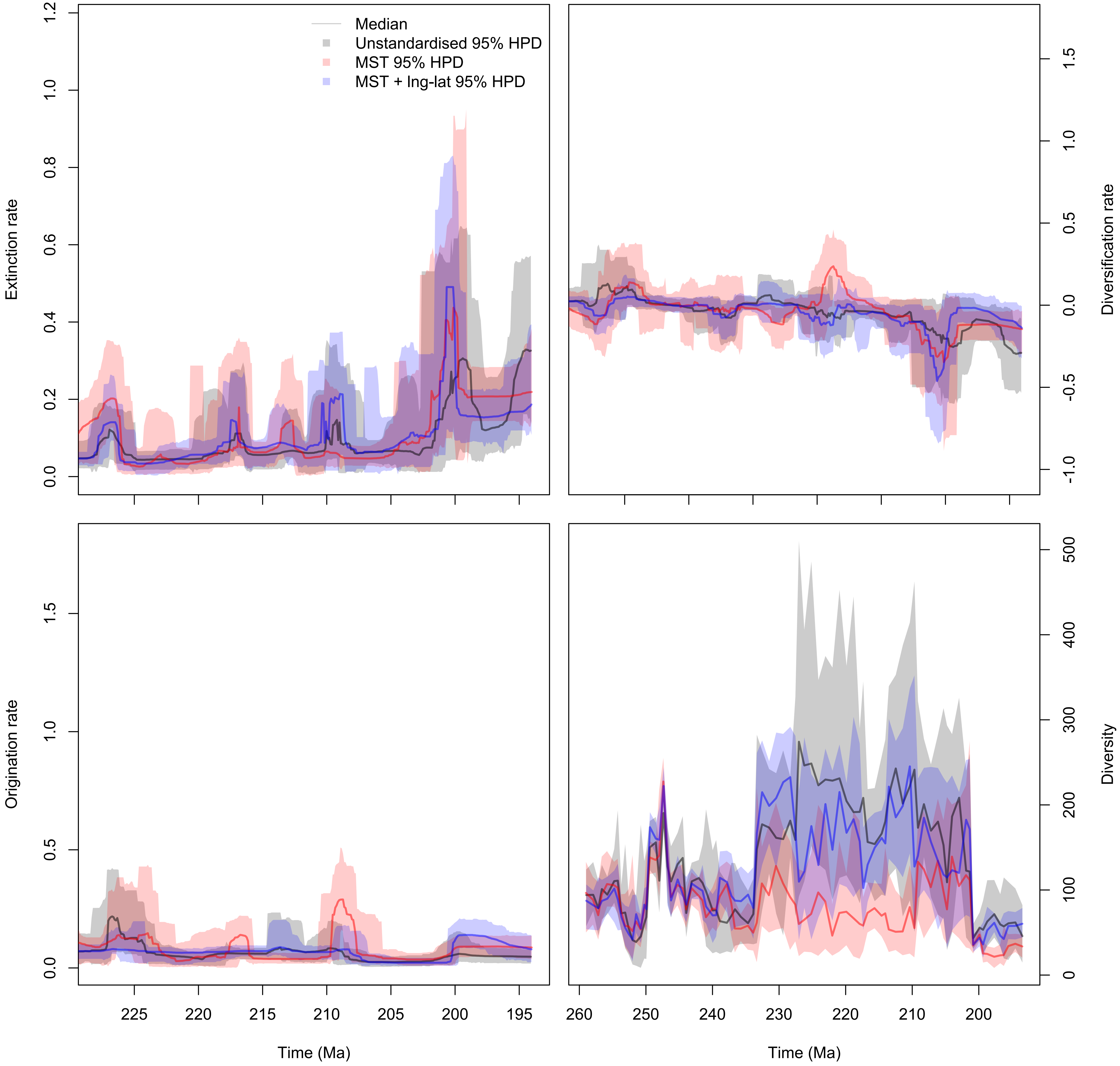

Supplementary Figure 40. Comparison of probabilistic origination, extinction and origination rates, and diversity under each data standardisation treatment for the North Panthalassic region

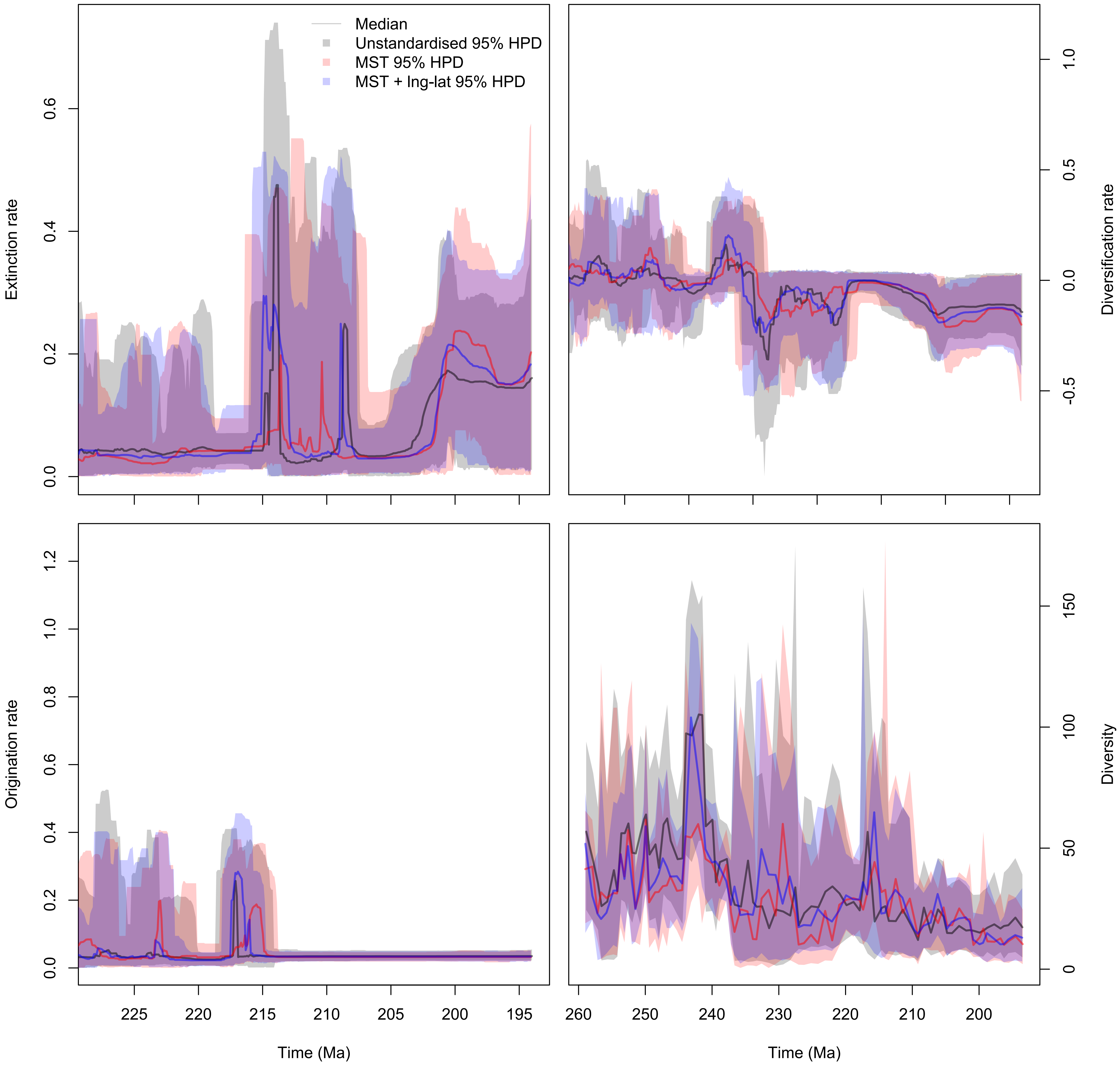

Supplementary Figure 41. Comparison of probabilistic origination, extinction and origination rates, and diversity under each data standardisation treatment for the Tangaroan region

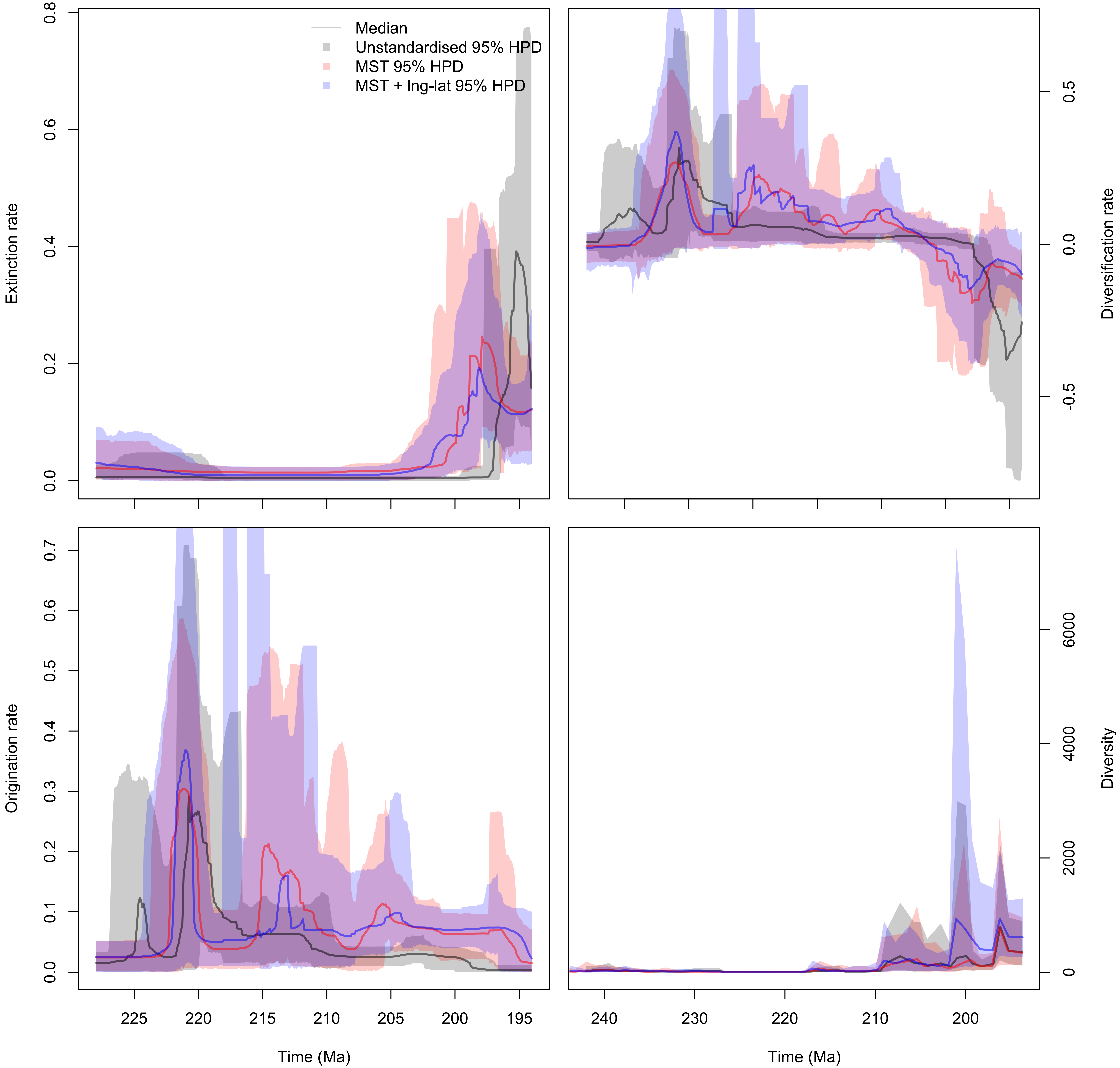

Supplementary Figure 42. Comparison of probabilistic origination, extinction and origination rates, and diversity under each data standardisation treatment for the South Panthalassic region

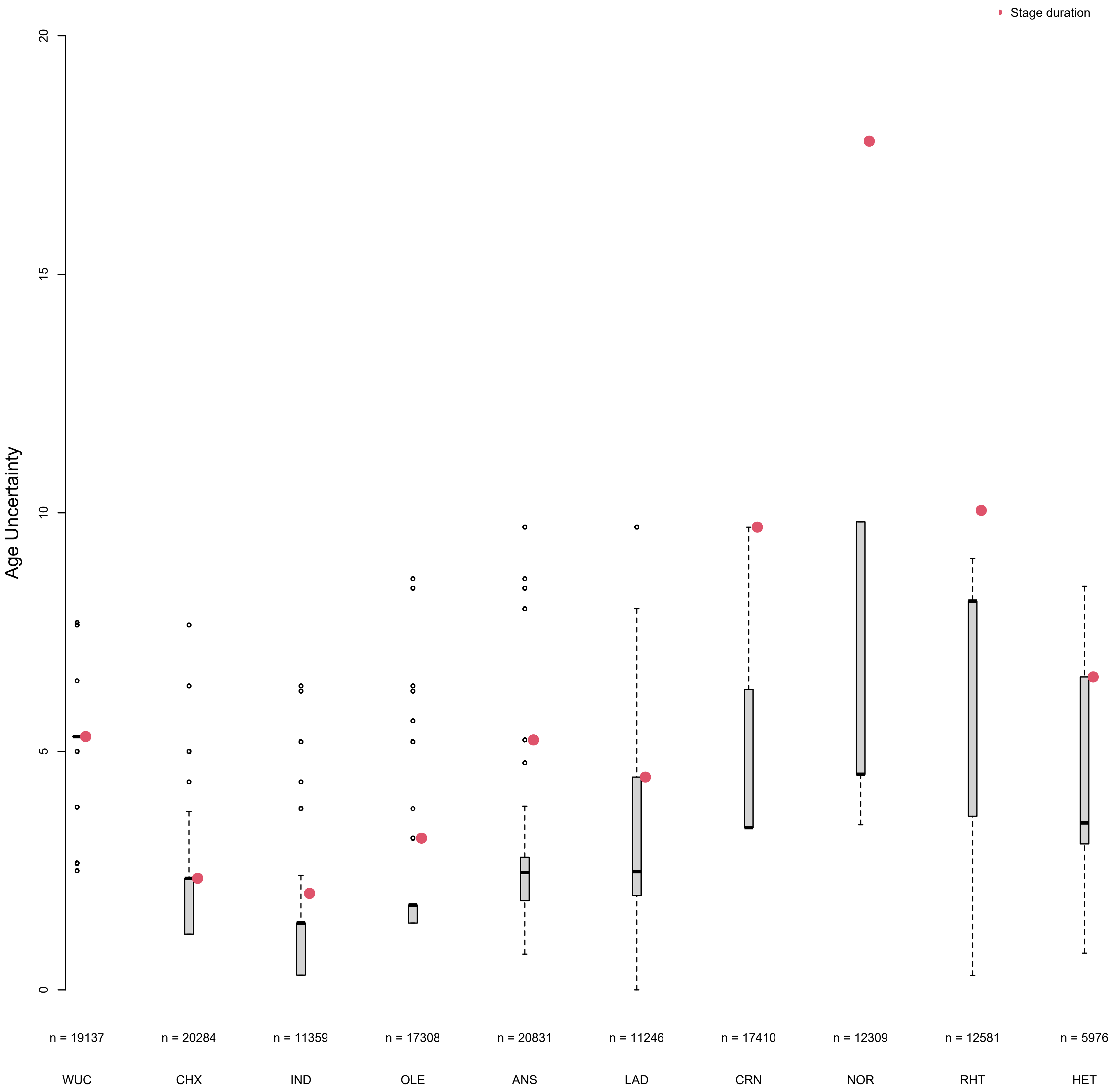

Stage duration
Age Uncertainty
0
5
10
15
20
n = 19137
n = 20284
n = 11359
n = 17308
n = 20831
n = 11246
n = 17410
n = 12309
n = 12581
n = 5976
WUC
CHX
IND
OLE
ANS
LAD
CRN
NOR
RHT
HET

Supplementary Tables 1-4: Pearson correlation between spatial extent and sampling-standardised diversity for Circumtethys (coverage q = 40%, 50%, 60% and 70%)

Unstandardised

	cor.coef_q4	p_q4	cor.coef_q5	p_q5	cor.coef_q6	p_q6	cor.coef_q7	p_q7
MST length (km)	0.344	0.165	0.348	0.162	0.351	0.16	0.356	0.156
Latitude range (deg)	-0.241	0.749	-0.247	0.754	-0.258	0.764	-0.275	0.779
Longitude range (deg)	0.605	**0.032**	0.601	**0.033**	0.592	**0.036**	0.575	**0.041**

MST

	cor.coef_q4	p_q4	cor.coef_q5	p_q5	cor.coef_q6	p_q6	cor.coef_q7	p_q7
MST length (km)	-0.605	0.968	-0.613	0.97	-0.619	0.972	-0.626	0.974
Latitude range (deg)	-0.272	0.776	-0.278	0.782	-0.292	0.793	-0.314	0.812
Longitude range (deg)	0.623	**0.027**	0.619	**0.028**	0.614	**0.03**	0.600	**0.033**

Lng-lat

	cor.coef_q4	p_q4	cor.coef_q5	p_q5	cor.coef_q6	p_q6	cor.coef_q7	p_q7
MST length (km)	0.351	0.160	0.359	0.154	0.365	0.150	0.375	0.143
Latitude range (deg)	0.145	0.344	0.146	0.344	0.138	0.352	0.124	0.367
Longitude range (deg)	0.307	0.194	0.296	0.203	0.283	0.214	0.266	0.228

Both

	cor.coef_q4	p_q4	cor.coef_q5	p_q5	cor.coef_q6	p_q6	cor.coef_q7	p_q7
MST length (km)	0.257	0.237	0.258	0.236	0.261	0.233	0.268	0.227
Latitude range (deg)	0.074	0.419	0.073	0.420	0.066	0.428	0.049	0.446
Longitude range (deg)	0.356	0.156	0.341	0.167	0.328	0.177	0.310	0.192

Supplementary Tables 5-8: Pearson correlation between spatial extent and sampling-standardised diversity for West Circumtethys (coverage q = 40%, 50%, 60% and 70%)

Unstandardised

	cor.coef_q4	p_q4	cor.coef_q5	p_q5	cor.coef_q6	p_q6	cor.coef_q7	p_q7
MST length (km)	0.843	**0.001**	0.848	**0.001**	0.851	**0.001**	0.852	**0.001**
Latitude range (deg)	0.265	0.23	0.267	0.228	0.268	0.227	0.271	0.224
Longitude range (deg)	0.519	0.062	0.511	0.066	0.506	0.068	0.505	0.068

MST

	cor.coef_q4	p_q4	cor.coef_q5	p_q5	cor.coef_q6	p_q6	cor.coef_q7	p_q7
MST length (km)	0.237	0.255	0.250	0.243	0.253	0.241	0.243	0.249
Latitude range (deg)	0.260	0.234	0.262	0.232	0.262	0.232	0.264	0.230
Longitude range (deg)	0.517	0.063	0.511	0.066	0.503	0.069	0.500	0.070

Lng-lat

	cor.coef_q4	p_q4	cor.coef_q5	p_q5	cor.coef_q6	p_q6	cor.coef_q7	p_q7
MST length (km)	0.690	**0.014**	0.700	**0.012**	0.708	**0.011**	0.708	**0.011**
Latitude range (deg)	0.226	0.265	0.228	0.264	0.227	0.264	0.228	0.264
Longitude range (deg)	0.263	0.231	0.261	0.233	0.259	0.235	0.263	0.232

Both

	cor.coef_q4	p_q4	cor.coef_q5	p_q5	cor.coef_q6	p_q6	cor.coef_q7	p_q7
MST length (km)	0.302	0.198	0.295	0.204	0.291	0.207	0.288	0.210
Latitude range (deg)	0.226	0.266	0.226	0.265	0.225	0.266	0.225	0.266
Longitude range (deg)	0.262	0.232	0.259	0.235	0.257	0.237	0.260	0.234

Supplementary Tables 9-12: Pearson correlation between spatial extent and sampling-standardised diversity for East Circumtethys (coverage q = 40%, 50%, 60% and 70%)

Unstandardised

	cor.coef_q4	p_q4	cor.coef_q5	p_q5	cor.coef_q6	p_q6	cor.coef_q7	p_q7
MST length (km)	0.089	0.404	0.085	0.408	0.077	0.416	0.077	0.417
Latitude range (deg)	-0.108	0.617	-0.126	0.636	-0.157	0.667	-0.192	0.702
Longitude range (deg)	-0.035	0.539	-0.033	0.536	-0.047	0.551	-0.069	0.576

MST

	cor.coef_q4	p_q4	cor.coef_q5	p_q5	cor.coef_q6	p_q6	cor.coef_q7	p_q7
MST length (km)	0.129	0.361	0.178	0.311	0.196	0.293	0.199	0.291
Latitude range (deg)	-0.206	0.716	-0.236	0.745	-0.275	0.779	-0.318	0.815
Longitude range (deg)	-0.187	0.697	-0.184	0.695	-0.203	0.713	-0.231	0.739

Lng-lat

	cor.coef_q4	p_q4	cor.coef_q5	p_q5	cor.coef_q6	p_q6	cor.coef_q7	p_q7
MST length (km)	0.137	0.353	0.130	0.36	0.121	0.369	0.116	0.374
Latitude range (deg)	-0.047	0.552	-0.064	0.57	-0.097	0.605	-0.138	0.648
Longitude range (deg)	-0.013	0.515	-0.018	0.52	-0.033	0.537	-0.055	0.560

Both

	cor.coef_q4	p_q4	cor.coef_q5	p_q5	cor.coef_q6	p_q6	cor.coef_q7	p_q7
MST length (km)	0.535	0.056	0.549	0.050	0.556	**0.047**	0.566	**0.044**
Latitude range (deg)	-0.125	0.635	-0.149	0.659	-0.189	0.699	-0.235	0.743
Longitude range (deg)	-0.259	0.765	-0.267	0.772	-0.286	0.788	-0.308	0.807

Supplementary Tables 13-16: Pearson correlation between spatial extent and sampling-standardised diversity for Boreal (coverage q = 40%, 50%, 60% and 70%)

Unstandardised

	cor.coef_q4	p_q4	cor.coef_q5	p_q5	cor.coef_q6	p_q6	cor.coef_q7	p_q7
MST length (km)	0.688	**0.014**	0.692	**0.013**	0.700	**0.012**	0.716	**0.01**
Latitude range (deg)	-0.213	0.722	-0.212	0.721	-0.215	0.724	-0.214	0.724
Longitude range (deg)	-0.264	0.769	-0.266	0.771	-0.271	0.776	-0.275	0.779

MST

	cor.coef_q4	p_q4	cor.coef_q5	p_q5	cor.coef_q6	p_q6	cor.coef_q7	p_q7
MST length (km)	0.391	0.132	0.413	0.118	0.447	0.098	0.507	0.068
Latitude range (deg)	-0.001	0.501	0.015	0.484	0.000	0.500	-0.043	0.547
Longitude range (deg)	-0.389	0.867	-0.387	0.865	-0.395	0.871	-0.382	0.862

Lng-lat

	cor.coef_q4	p_q4	cor.coef_q5	p_q5	cor.coef_q6	p_q6	cor.coef_q7	p_q7
MST length (km)	0.742	**0.007**	0.743	**0.007**	0.752	**0.006**	0.765	**0.005**
Latitude range (deg)	0.036	0.461	0.033	0.464	0.036	0.46	0.038	0.458
Longitude range (deg)	0.334	0.173	0.308	0.193	0.282	0.215	0.257	0.237

Both

	cor.coef_q4	p_q4	cor.coef_q5	p_q5	cor.coef_q6	p_q6	cor.coef_q7	p_q7
MST length (km)	0.604	**0.032**	0.578	**0.04**	0.581	**0.039**	0.584	**0.038**
Latitude range (deg)	0.003	0.496	0.000	0.5	0.008	0.492	0.008	0.491
Longitude range (deg)	0.253	0.241	0.238	0.254	0.213	0.277	0.181	0.309

Supplementary Tables 17-20: Pearson correlation between spatial extent and sampling-standardised diversity for North Panthalassic-short (coverage q = 40%, 50%, 60% and 70%)

Unstandardised

	cor.coef_q4	p_q4	cor.coef_q5	p_q5	cor.coef_q6	p_q6	cor.coef_q7	p_q7
MST length (km)	0.565	0.056	0.580	0.051	0.600	**0.044**	0.616	**0.039**
Latitude range (deg)	0.481	0.095	0.490	0.090	0.504	0.083	0.510	0.08

	cor.coef_q4	p_q4	cor.coef_q5	p_q5	cor.coef_q6	p_q6	cor.coef_q7	p_q7
Longitude range (deg)	-0.035	0.536	-0.022	0.522	-0.003	0.503	0.020	0.48

MST

	cor.coef_q4	p_q4	cor.coef_q5	p_q5	cor.coef_q6	p_q6	cor.coef_q7	p_q7
MST length (km)	0.196	0.307	0.203	0.300	0.219	0.285	0.236	0.271
Latitude range (deg)	0.444	0.115	0.457	0.108	0.473	0.100	0.486	0.092
Longitude range (deg)	-0.066	0.567	-0.050	0.550	-0.027	0.528	0.002	0.498

Lng-lat

	cor.coef_q4	p_q4	cor.coef_q5	p_q5	cor.coef_q6	p_q6	cor.coef_q7	p_q7
MST length (km)	0.756	**0.009**	0.769	**0.008**	0.781	**0.006**	0.793	**0.005**
Latitude range (deg)	0.546	0.064	0.561	0.058	0.577	0.052	0.595	**0.046**
Longitude range (deg)	0.114	0.385	0.133	0.366	0.160	0.34	0.184	0.317

Both

	cor.coef_q4	p_q4	cor.coef_q5	p_q5	cor.coef_q6	p_q6	cor.coef_q7	p_q7
MST length (km)	0.612	**0.04**	0.631	**0.034**	0.652	**0.029**	0.673	**0.023**
Latitude range (deg)	0.541	0.066	0.558	0.059	0.576	0.052	0.595	**0.045**
Longitude range (deg)	0.114	0.385	0.132	0.367	0.159	0.341	0.183	0.319

Supplementary Tables 21-24: Pearson correlation between spatial extent and sampling-standardised diversity for North Panthalassic (coverage q = 40%, 50%, 60% and 70%)

Unstandardised

	cor.coef_q4	p_q4	cor.coef_q5	p_q5	cor.coef_q6	p_q6	cor.coef_q7	p_q7
MST length (km)	0.599	**0.034**	0.608	**0.031**	0.620	**0.028**	0.628	**0.026**
Latitude range (deg)	0.510	0.066	0.512	0.065	0.516	0.063	0.515	0.064
Longitude range (deg)	0.155	0.334	0.162	0.327	0.173	0.317	0.184	0.305

MST

	cor.coef_q4	p_q4	cor.coef_q5	p_q5	cor.coef_q6	p_q6	cor.coef_q7	p_q7
MST length (km)	0.342	0.167	0.343	0.166	0.348	0.162	0.352	0.159
Latitude range (deg)	0.490	0.075	0.494	0.073	0.498	0.071	0.500	0.070
Longitude range (deg)	0.137	0.353	0.146	0.344	0.158	0.332	0.174	0.315

Lng-lat

	cor.coef_q4	p_q4	cor.coef_q5	p_q5	cor.coef_q6	p_q6	cor.coef_q7	p_q7
MST length (km)	0.756	**0.006**	0.767	**0.005**	0.777	**0.004**	0.786	**0.004**
Latitude range (deg)	0.506	0.068	0.515	0.064	0.523	0.06	0.530	0.058
Longitude range (deg)	0.242	0.251	0.251	0.242	0.264	0.231	0.272	0.224

Both

	cor.coef_q4	p_q4	cor.coef_q5	p_q5	cor.coef_q6	p_q6	cor.coef_q7	p_q7
MST length (km)	0.618	**0.028**	0.634	**0.025**	0.650	**0.021**	0.665	**0.018**
Latitude range (deg)	0.502	0.07	0.512	0.065	0.522	0.061	0.530	0.057
Longitude range (deg)	0.240	0.252	0.250	0.243	0.263	0.232	0.271	0.224

Supplementary Tables 25-28: Pearson correlation between spatial extent and sampling-standardised diversity for Tangaroan (coverage q = 40%, 50%, 60% and 70%)

Unstandardised

	cor.coef_q4	p_q4	cor.coef_q5	p_q5	cor.coef_q6	p_q6	cor.coef_q7	p_q7
MST length (km)	-0.247	0.754	-0.122	0.631	0.076	0.417	0.317	0.186
Latitude range (deg)	-0.079	0.585	-0.070	0.576	-0.024	0.527	0.035	0.462
Longitude range (deg)	0.514	0.064	0.504	0.069	0.427	0.109	0.274	0.222

MST

	cor.coef_q4	p_q4	cor.coef_q5	p_q5	cor.coef_q6	p_q6	cor.coef_q7	p_q7
MST length (km)	-0.321	0.817	-0.246	0.753	-0.123	0.632	0.039	0.458
Latitude range (deg)	-0.125	0.634	-0.123	0.632	-0.116	0.625	-0.082	0.589
Longitude range (deg)	0.512	0.065	0.493	0.074	0.414	0.117	0.265	0.230

Lng-lat

	cor.coef_q4	p_q4	cor.coef_q5	p_q5	cor.coef_q6	p_q6	cor.coef_q7	p_q7
MST length (km)	-0.187	0.697	-0.053	0.558	0.177	0.313	0.410	0.120
Latitude range (deg)	0.472	0.084	0.492	0.074	0.484	0.078	0.416	0.116
Longitude range (deg)	0.585	**0.038**	0.622	**0.028**	0.570	**0.043**	0.442	0.100

Both

	cor.coef_q4	p_q4	cor.coef_q5	p_q5	cor.coef_q6	p_q6	cor.coef_q7	p_q7
MST length (km)	-0.250	0.757	-0.147	0.658	0.042	0.455	0.285	0.212
Latitude range (deg)	0.479	0.081	0.490	0.075	0.474	0.083	0.373	0.145
Longitude range (deg)	0.580	**0.039**	0.626	**0.026**	0.596	**0.035**	0.464	0.089

Supplementary Tables 29-32: Pearson correlation between spatial extent and sampling-standardised diversity for South Panthalassic (coverage q = 40%, 50%, 60% and 70%)

Unstandardised

	cor.coef_q4	p_q4	cor.coef_q5	p_q5	cor.coef_q6	p_q6	cor.coef_q7	p_q7
MST length (km)	-0.806	0.950	-0.792	0.945	-0.746	0.926	-0.651	0.883
Latitude range (deg)	-0.949	0.998	-0.947	0.998	-0.926	0.996	-0.874	0.989
Longitude range (deg)	-0.924	0.996	-0.927	0.996	-0.905	0.993	-0.849	0.984

MST

	cor.coef_q4	p_q4	cor.coef_q5	p_q5	cor.coef_q6	p_q6	cor.coef_q7	p_q7
MST length (km)	-0.944	0.992	-0.940	0.991	-0.920	0.986	-0.878	0.975
Latitude range (deg)	-0.950	0.998	-0.948	0.998	-0.936	0.997	-0.909	0.994
Longitude range (deg)	-0.938	0.997	-0.940	0.997	-0.929	0.996	-0.900	0.993

Lng-lat

	cor.coef_q4	p_q4	cor.coef_q5	p_q5	cor.coef_q6	p_q6	cor.coef_q7	p_q7
MST length (km)	-0.633	0.874	-0.792	0.945	-0.861	0.970	-0.853	0.967
Latitude range (deg)	-0.576	0.884	-0.692	0.936	-0.756	0.959	-0.785	0.968
Longitude range (deg)	-0.583	0.888	-0.704	0.941	-0.769	0.963	-0.795	0.971

Both

	cor.coef_q4	p_q4	cor.coef_q5	p_q5	cor.coef_q6	p_q6	cor.coef_q7	p_q7
MST length (km)	-0.467	0.786	-0.608	0.862	-0.681	0.897	-0.720	0.915
Latitude range (deg)	-0.568	0.880	-0.654	0.921	-0.699	0.939	-0.723	0.948
Longitude range (deg)	-0.583	0.888	-0.673	0.928	-0.719	0.946	-0.743	0.955

Supplementary Tables 33-36: Spearman correlation between spatial extent and sampling-standardised diversity for Circumtethys (coverage q = 40%, 50%, 60% and 70%)

Unstandardised

	cor.coef_q4	p_q4	cor.coef_q5	p_q5	cor.coef_q6	p_q6	cor.coef_q7	p_q7
MST length (km)	0.139	0.354	0.139	0.354	0.139	0.354	0.224	0.268
Latitude range (deg)	-0.406	0.884	-0.406	0.884	-0.406	0.884	-0.370	0.861
Longitude range (deg)	0.600	**0.037**	0.600	**0.037**	0.600	**0.037**	0.552	0.052

MST

	cor.coef_q4	p_q4	cor.coef_q5	p_q5	cor.coef_q6	p_q6	cor.coef_q7	p_q7
MST length (km)	-0.576	0.96	-0.661	0.98	-0.661	0.98	-0.685	0.984
Latitude range (deg)	-0.467	0.917	-0.539	0.948	-0.539	0.948	-0.552	0.952
Longitude range (deg)	0.709	**0.014**	0.721	**0.012**	0.721	**0.012**	0.697	**0.016**

Lng-lat

	cor.coef_q4	p_q4	cor.coef_q5	p_q5	cor.coef_q6	p_q6	cor.coef_q7	p_q7
MST length (km)	0.200	0.292	0.127	0.366	0.248	0.246	0.309	0.194
Latitude range (deg)	0.091	0.406	0.164	0.328	0.152	0.341	0.079	0.419
Longitude range (deg)	0.188	0.304	0.285	0.214	0.176	0.316	0.115	0.379

Both

	cor.coef_q4	p_q4	cor.coef_q5	p_q5	cor.coef_q6	p_q6	cor.coef_q7	p_q7
MST length (km)	-0.103	0.621	-0.079	0.594	-0.079	0.594	-0.079	0.594
Latitude range (deg)	-0.212	0.732	-0.248	0.765	-0.248	0.765	-0.248	0.765
Longitude range (deg)	0.382	0.139	0.358	0.156	0.358	0.156	0.358	0.156

Supplementary Tables 37-40: Spearman correlation between spatial extent and sampling-standardised diversity for West Circumtethys (coverage q = 40%, 50%, 60% and 70%)

Unstandardised

	cor.coef_q4	p_q4	cor.coef_q5	p_q5	cor.coef_q6	p_q6	cor.coef_q7	p_q7
MST length (km)	0.770	**0.007**	0.758	**0.008**	0.758	**0.008**	0.758	**0.008**
Latitude range (deg)	0.152	0.341	0.188	0.304	0.188	0.304	0.188	0.304
Longitude range (deg)	0.442	0.102	0.358	0.156	0.358	0.156	0.358	0.156

MST

	cor.coef_q4	p_q4	cor.coef_q5	p_q5	cor.coef_q6	p_q6	cor.coef_q7	p_q7
MST length (km)	0.042	0.459	0.042	0.459	0.042	0.459	0.042	0.459
Latitude range (deg)	0.188	0.304	0.188	0.304	0.188	0.304	0.188	0.304
Longitude range (deg)	0.358	0.156	0.358	0.156	0.358	0.156	0.358	0.156

Lng-lat

	cor.coef_q4	p_q4	cor.coef_q5	p_q5	cor.coef_q6	p_q6	cor.coef_q7	p_q7
MST length (km)	0.661	**0.022**	0.661	**0.022**	0.661	**0.022**	0.624	**0.03**
Latitude range (deg)	0.103	0.393	0.103	0.393	0.103	0.393	0.042	0.459
Longitude range (deg)	0.280	0.217	0.280	0.217	0.280	0.217	0.292	0.207

Both

	cor.coef_q4	p_q4	cor.coef_q5	p_q5	cor.coef_q6	p_q6	cor.coef_q7	p_q7
MST length (km)	0.297	0.203	0.297	0.203	0.297	0.203	0.297	0.203
Latitude range (deg)	0.055	0.446	0.055	0.446	0.055	0.446	0.055	0.446
Longitude range (deg)	0.328	0.177	0.328	0.177	0.328	0.177	0.328	0.177

Supplementary Tables 41-44: Spearman correlation between spatial extent and sampling-standardised diversity for East Circumtethys (coverage q = 40%, 50%, 60% and 70%)

Unstandardised

	cor.coef_q4	p_q4	cor.coef_q5	p_q5	cor.coef_q6	p_q6	cor.coef_q7	p_q7
MST length (km)	0.248	0.246	0.261	0.235	0.261	0.235	0.248	0.246
Latitude range (deg)	-0.176	0.696	-0.164	0.684	-0.164	0.684	-0.176	0.696
Longitude range (deg)	0.139	0.354	0.176	0.316	0.176	0.316	0.139	0.354

MST

	cor.coef_q4	p_q4	cor.coef_q5	p_q5	cor.coef_q6	p_q6	cor.coef_q7	p_q7
MST length (km)	0.018	0.486	0.042	0.459	0.030	0.473	0.018	0.486
Latitude range (deg)	-0.188	0.708	-0.176	0.696	-0.164	0.684	-0.224	0.743
Longitude range (deg)	0.030	0.473	0.055	0.446	0.030	0.473	-0.079	0.594

Lng-lat

	cor.coef_q4	p_q4	cor.coef_q5	p_q5	cor.coef_q6	p_q6	cor.coef_q7	p_q7
MST length (km)	0.164	0.328	0.164	0.328	0.164	0.328	0.164	0.328
Latitude range (deg)	0.030	0.473	0.030	0.473	0.030	0.473	0.030	0.473
Longitude range (deg)	-0.067	0.581	-0.067	0.581	-0.067	0.581	-0.067	0.581

Both

	cor.coef_q4	p_q4	cor.coef_q5	p_q5	cor.coef_q6	p_q6	cor.coef_q7	p_q7
MST length (km)	0.418	0.116	0.345	0.165	0.382	0.139	0.370	0.148
Latitude range (deg)	0.103	0.393	0.079	0.419	0.067	0.432	-0.030	0.541
Longitude range (deg)	-0.333	0.835	-0.479	0.923	-0.406	0.884	-0.382	0.869

Supplementary Tables 45-48: Spearman correlation between spatial extent and sampling-standardised diversity for Boreal (coverage q = 40%, 50%, 60% and 70%)

Unstandardised

	cor.coef_q4	p_q4	cor.coef_q5	p_q5	cor.coef_q6	p_q6	cor.coef_q7	p_q7
MST length (km)	0.782	**0.006**	0.782	**0.006**	0.782	**0.006**	0.806	**0.004**
Latitude range (deg)	-0.152	0.672	-0.224	0.743	-0.236	0.754	-0.273	0.786
Longitude range (deg)	-0.467	0.917	-0.418	0.891	-0.479	0.923	-0.394	0.876

MST

	cor.coef_q4	p_q4	cor.coef_q5	p_q5	cor.coef_q6	p_q6	cor.coef_q7	p_q7
MST length (km)	0.176	0.316	0.127	0.366	0.164	0.328	0.285	0.214
Latitude range (deg)	-0.152	0.672	-0.200	0.720	-0.212	0.732	-0.309	0.816
Longitude range (deg)	-0.394	0.876	-0.358	0.852	-0.479	0.923	-0.418	0.891

Lng-lat

	cor.coef_q4	p_q4	cor.coef_q5	p_q5	cor.coef_q6	p_q6	cor.coef_q7	p_q7
MST length (km)	0.770	**0.007**	0.733	**0.011**	0.733	**0.011**	0.745	**0.009**
Latitude range (deg)	-0.079	0.594	-0.176	0.696	-0.176	0.696	-0.212	0.732
Longitude range (deg)	0.467	0.089	0.418	0.116	0.418	0.116	0.406	0.124

Both

	cor.coef_q4	p_q4	cor.coef_q5	p_q5	cor.coef_q6	p_q6	cor.coef_q7	p_q7
MST length (km)	0.321	0.184	0.455	0.095	0.442	0.102	0.442	0.102
Latitude range (deg)	-0.079	0.594	-0.127	0.646	-0.139	0.659	-0.200	0.720
Longitude range (deg)	0.115	0.379	-0.006	0.514	0.018	0.486	0.006	0.500

Supplementary Tables 49-52: Spearman correlation between spatial extent and sampling-standardised diversity for North Panthalassic-short (coverage q = 40%, 50%, 60% and 70%)

Unstandardised

	cor.coef_q4	p_q4	cor.coef_q5	p_q5	cor.coef_q6	p_q6	cor.coef_q7	p_q7
MST length (km)	0.250	0.260	0.250	0.260	0.417	0.135	0.333	0.193
Latitude range (deg)	0.383	0.156	0.383	0.156	0.533	0.074	0.433	0.125
Longitude range (deg)	-0.050	0.560	-0.050	0.560	-0.083	0.595	0.000	0.509

MST

	cor.coef_q4	p_q4	cor.coef_q5	p_q5	cor.coef_q6	p_q6	cor.coef_q7	p_q7
MST length (km)	-0.033	0.544	-0.033	0.544	-0.033	0.544	-0.033	0.544
Latitude range (deg)	0.383	0.156	0.383	0.156	0.383	0.156	0.383	0.156
Longitude range (deg)	-0.050	0.560	-0.050	0.560	-0.050	0.560	-0.050	0.560

Lng-lat

	cor.coef_q4	p_q4	cor.coef_q5	p_q5	cor.coef_q6	p_q6	cor.coef_q7	p_q7
MST length (km)	0.60	**0.048**	0.60	**0.048**	0.60	**0.048**	0.60	**0.048**
Latitude range (deg)	0.55	0.066	0.55	0.066	0.55	0.066	0.55	0.066
Longitude range (deg)	-0.10	0.612	-0.10	0.612	-0.10	0.612	-0.10	0.612

Both

	cor.coef_q4	p_q4	cor.coef_q5	p_q5	cor.coef_q6	p_q6	cor.coef_q7	p_q7
MST length (km)	0.60	**0.048**	0.60	**0.048**	0.60	**0.048**	0.60	**0.048**
Latitude range (deg)	0.55	0.066	0.55	0.066	0.55	0.066	0.55	0.066
Longitude range (deg)	-0.10	0.612	-0.10	0.612	-0.10	0.612	-0.10	0.612

Supplementary Tables 53-56: Spearman correlation between spatial extent and sampling-standardised diversity for North Panthalassic (coverage q = 40%, 50%, 60% and 70%)

Unstandardised

	cor.coef_q4	p_q4	cor.coef_q5	p_q5	cor.coef_q6	p_q6	cor.coef_q7	p_q7
MST length (km)	0.455	0.095	0.455	0.095	0.576	**0.044**	0.515	0.066
Latitude range (deg)	0.552	0.052	0.552	0.052	0.661	**0.022**	0.588	**0.04**
Longitude range (deg)	0.236	0.257	0.236	0.257	0.212	0.28	0.273	0.224

MST

	cor.coef_q4	p_q4	cor.coef_q5	p_q5	cor.coef_q6	p_q6	cor.coef_q7	p_q7
MST length (km)	0.164	0.328	0.164	0.328	0.164	0.328	0.164	0.328
Latitude range (deg)	0.455	0.095	0.455	0.095	0.455	0.095	0.455	0.095
Longitude range (deg)	0.200	0.292	0.200	0.292	0.200	0.292	0.200	0.292

Lng-lat

	cor.coef_q4	p_q4	cor.coef_q5	p_q5	cor.coef_q6	p_q6	cor.coef_q7	p_q7
MST length (km)	0.600	**0.037**	0.600	**0.037**	0.600	**0.037**	0.564	**0.048**
Latitude range (deg)	0.564	**0.048**	0.564	**0.048**	0.564	**0.048**	0.527	0.061
Longitude range (deg)	0.018	0.486	0.018	0.486	0.018	0.486	-0.018	0.527

Both

	cor.coef_q4	p_q4	cor.coef_q5	p_q5	cor.coef_q6	p_q6	cor.coef_q7	p_q7
MST length (km)	0.600	**0.037**	0.600	**0.037**	0.600	**0.037**	0.564	**0.048**
Latitude range (deg)	0.564	**0.048**	0.564	**0.048**	0.564	**0.048**	0.527	0.061
Longitude range (deg)	0.018	0.486	0.018	0.486	0.018	0.486	-0.018	0.527

Supplementary Tables 57-60: Spearman correlation between spatial extent and sampling-standardised diversity for Tangaroan (coverage q = 40%, 50%, 60% and 70%)

Unstandardised

	cor.coef_q4	p_q4	cor.coef_q5	p_q5	cor.coef_q6	p_q6	cor.coef_q7	p_q7
MST length (km)	-0.224	0.743	-0.200	0.720	0.067	0.432	0.370	0.148
Latitude range (deg)	-0.103	0.621	-0.139	0.659	-0.164	0.684	0.042	0.459
Longitude range (deg)	0.382	0.139	0.345	0.165	0.285	0.214	0.273	0.224

MST

	cor.coef_q4	p_q4	cor.coef_q5	p_q5	cor.coef_q6	p_q6	cor.coef_q7	p_q7
MST length (km)	-0.127	0.646	-0.127	0.646	0.127	0.366	0.152	0.341
Latitude range (deg)	-0.200	0.720	-0.200	0.720	-0.236	0.754	-0.224	0.743
Longitude range (deg)	0.345	0.165	0.345	0.165	0.321	0.184	0.309	0.194

Lng-lat

	cor.coef_q4	p_q4	cor.coef_q5	p_q5	cor.coef_q6	p_q6	cor.coef_q7	p_q7
MST length (km)	-0.164	0.684	-0.091	0.607	0.212	0.280	0.345	0.165
Latitude range (deg)	0.358	0.156	0.261	0.235	0.261	0.235	0.261	0.235
Longitude range (deg)	0.612	**0.033**	0.539	0.057	0.491	0.077	0.394	0.131

Both

	cor.coef_q4	p_q4	cor.coef_q5	p_q5	cor.coef_q6	p_q6	cor.coef_q7	p_q7
MST length (km)	-0.164	0.684	-0.164	0.684	0.018	0.486	0.285	0.214
Latitude range (deg)	0.406	0.124	0.370	0.148	0.333	0.174	0.261	0.235
Longitude range (deg)	0.564	**0.048**	0.612	**0.033**	0.503	0.072	0.394	0.131

Supplementary Tables 61-64: Spearman correlation between spatial extent and sampling-standardised diversity for South Panthalassic (coverage q = 40%, 50%, 60% and 70%)

Unstandardised

	cor.coef_q4	p_q4	cor.coef_q5	p_q5	cor.coef_q6	p_q6	cor.coef_q7	p_q7
MST length (km)	-0.900	0.992	-0.900	0.992	-0.900	0.992	-0.900	0.992
Latitude range (deg)	-0.886	0.992	-0.886	0.992	-0.886	0.992	-0.886	0.992
Longitude range (deg)	-1.000	1.000	-1.000	1.000	-1.000	1.000	-1.000	1.000

MST

	cor.coef_q4	p_q4	cor.coef_q5	p_q5	cor.coef_q6	p_q6	cor.coef_q7	p_q7
MST length (km)	-1.000	1.000	-1.000	1.000	-1.000	1.000	-1.000	1.000
Latitude range (deg)	-0.886	0.992	-0.886	0.992	-0.886	0.992	-0.886	0.992
Longitude range (deg)	-1.000	1.000	-1.000	1.000	-1.000	1.000	-1.000	1.000

Lng-lat

	cor.coef_q4	p_q4	cor.coef_q5	p_q5	cor.coef_q6	p_q6	cor.coef_q7	p_q7
MST length (km)	-0.400	0.775	-0.700	0.933	-0.900	0.992	-0.900	0.992
Latitude range (deg)	-0.314	0.751	-0.486	0.851	-0.714	0.949	-0.714	0.949
Longitude range (deg)	-0.429	0.822	-0.657	0.932	-0.829	0.983	-0.829	0.983

Both

	cor.coef_q4	p_q4	cor.coef_q5	p_q5	cor.coef_q6	p_q6	cor.coef_q7	p_q7
MST length (km)	0.000	0.525	-0.400	0.775	-0.700	0.933	-0.700	0.933
Latitude range (deg)	-0.314	0.751	-0.486	0.851	-0.714	0.949	-0.714	0.949
Longitude range (deg)	-0.429	0.822	-0.657	0.932	-0.829	0.983	-0.829	0.983

ID	Speciation rates	Speciation shifts	Extinction rates	Extinction shifts	Preservation rates	Lineage heterogeneity	Preservation shifts
A	0.4, 0.01	25	0.05, 0.01	25	0.25 - 2.5	-	23, 15, 8, 5.3, 2.6
B	0.4, 0.01	25	0.05, 0.01	25	0.25 - 2.5; 0*	-	23, 15, 8, 5.3, 2.6
C	0.4, 0.1, 0.01	20, 10	0.05, 0.3, 0.01	15, 10	0.25 - 2.5	-	23, 15, 8, 5.3, 2.6
D	0.4, 0.1, 0.01	20, 10	0.05, 0.3, 0.01	15, 10	0.25 - 2.5	0.5	23, 15, 8, 5.3, 2.6
E	0.2, 0.4	10	0.01, 0.9, 0.1	10, 7	0.25 - 2.5	-	23, 15, 8, 5.3, 2.6
F	0.2, 0.4	10	0.01, 0.9, 0.1	10, 7	0.25 - 2.5	0.5	23, 15, 8, 5.3, 2.6
