# Supplementary material for: Global diversity dynamics in the fossil record are regionally heterogeneous
Source: Nat Commun. 2022 May 18;13:2751. doi: 10.1038/s41467-022-30507-0 (PMC9117201; doi:10.1038/s41467-022-30507-0)
Supplement: Supplementary file 3 — Reporting Summary [file 41467_2022_30507_MOESM3_ESM.pdf]

nature portfolio

Corresponding author(s): Joseph T. Flannery-Sutherland, jf15558@bristol.ac.uk

Last updated by author(s): Apr 22, 2022

Reporting Summary

Nature Portfolio wishes to improve the reproducibility of the work that we publish. This form provides structure for consistency and transparency in reporting. For further information on Nature Portfolio policies, see our Editorial Policies and the Editorial Policy Checklist.

Statistics

For all statistical analyses, confirm that the following items are present in the figure legend, table legend, main text, or Methods section.

n/a	Confirmed	
☐	☒	The exact sample size (n) for each experimental group/condition, given as a discrete number and unit of measurement
☐	☒	A statement on whether measurements were taken from distinct samples or whether the same sample was measured repeatedly
☐	☒	The statistical test(s) used AND whether they are one- or two-sided<br>*Only common tests should be described solely by name; describe more complex techniques in the Methods section.*
☒	☐	A description of all covariates tested
☐	☒	A description of any assumptions or corrections, such as tests of normality and adjustment for multiple comparisons
☐	☒	A full description of the statistical parameters including central tendency (e.g. means) or other basic estimates (e.g. regression coefficient) AND variation (e.g. standard deviation) or associated estimates of uncertainty (e.g. confidence intervals)
☐	☒	For null hypothesis testing, the test statistic (e.g. F, t, r) with confidence intervals, effect sizes, degrees of freedom and P value noted<br>*Give P values as exact values whenever suitable.*
☐	☒	For Bayesian analysis, information on the choice of priors and Markov chain Monte Carlo settings
☒	☐	For hierarchical and complex designs, identification of the appropriate level for tests and full reporting of outcomes
☐	☒	Estimates of effect sizes (e.g. Cohen's d, Pearson's r), indicating how they were calculated

Our web collection on statistics for biologists contains articles on many of the points above.

Software and code

Policy information about availability of computer code

Data collection	No software was used for data collection
Data analysis	Most analyses were performed using R (v4.1) with scripts available in the electronic supplement. Other analyses were conducted using: PyRate, an open source software package available on Github (https://github.com/dsilvestro/PyRate); Tracer, a freeware package available on Github (https://github.com/beast-dev/tracer/releases/tag/v1.7.1); or a custom piece of software which is included in the electronic supplement and will be additionally made available on Github. R package versions: stringr, 1.4.0; dplyr, 1.0.8; data.table, 1.14.2; icosa, 0.10.1; hexbin, 1.28.2; sf, 1.0.7; sp, 1.4.7; geosphere, 1.5.14; maptools, 1.1.3; colorDF, 0.1.4; fields, 1.3.3; Orcs, 1.2.1; ape, 5.6.2; igraph, 1.3.1; GeoRange, 0.1.0; Rcpp, 1.0.8.3; curl, 4.3.2; knitr, 1.38; rmarkdown, 2.13; scales, 1.1.1; HDInterval. 0.2.2; vioplot, 0.3.7; iNEXT, 2.0.2; foreach, 1.5.2; doParallel, 1.0.17

For manuscripts utilizing custom algorithms or software that are central to the research but not yet described in published literature, software must be made available to editors and reviewers. We strongly encourage code deposition in a community repository (e.g. GitHub). See the Nature Portfolio guidelines for submitting code & software for further information.

Data

Policy information about availability of data

All manuscripts must include a data availability statement. This statement should provide the following information, where applicable:

- Accession codes, unique identifiers, or web links for publicly available datasets
- A description of any restrictions on data availability
- For clinical datasets or third party data, please ensure that the statement adheres to our policy

A description of data availability is included in the manuscript. The data itself is available in the electronic supplement

Field-specific reporting

Please select the one below that is the best fit for your research. If you are not sure, read the appropriate sections before making your selection.

☐ Life sciences ☐ Behavioural & social sciences ☒ Ecological, evolutionary & environmental sciences

For a reference copy of the document with all sections, see nature.com/documents/nr-reporting-summary-flat.pdf

Ecological, evolutionary & environmental sciences study design

All studies must disclose on these points even when the disclosure is negative.

Study description	This study presents a method to spatially standardise fossil occurrence datasets, then calculates diversity and diversification rates from standardised (four spatial standardisation treatments), regionalised (seven regions) Late Permian to Early Jurassic datasets using Bayesian methods. 10 age randomised replicates were analysed for each regional dataset
Research sample	Fossil occurrence data from the Paleobiology Database and the primary literature. These are the standard sources for macroevolutionary investigations of this sort and no other databases are available for this study interval. The sample represents all known marine fossil life in our study interval
Sampling strategy	Data was compiled to provide the largest sample size possible, excluding the small amount of data excluded during cleaning. This largest possible sample represents the empirical, accessible fossil record known to palaeobiologists. There is nothing larger. The sample provides global coverage and is a larger data sample than any previously analysed in the literature, all of which have been taken as sufficient for analysis of the kinds we perform
Data collection	Data was downloaded from the Palaeobiology Database and the primary literature using the curl R package to access the PBDB API. The data itself is a community upload effort spanning several decades and 10s of individuals. These data have been used by multiple studies previously
Timing and spatial scale	Data was downloaded once (no collection gaps) only only a global scale. Single downloads prior to analysis are commonplace for PBDB studies as significant data accumulations are unlikely to occur (this was checked during our analyses to be certain)
Data exclusions	Occurrences from minor clades (<1000 occurrences) was dropped as these represent noise from sporadically sampled groups which do not provide a reliable diversification signal, along with data with an age uncertainty >10 Ma (common practice in palaeobiologica studies as the PBDB is broadly at substage level for its occurrences). Data exclusion is fully documented in the methods
Reproducibility	All data and code needed to recreate the analyses are present in the electronic supplement
Randomization	Samples were divided into temporal bins based on their stratigraphic age. There were no associated covariates
Blinding	Blinding was not required by any of the data acquisition or analytical methods as there are no biases expected between treatments or samples

Did the study involve field work? ☐ Yes ☒ No

Reporting for specific materials, systems and methods

We require information from authors about some types of materials, experimental systems and methods used in many studies. Here, indicate whether each material, system or method listed is relevant to your study. If you are not sure if a list item applies to your research, read the appropriate section before selecting a response.

Materials & experimental systems

n/a	Involved in the study	
☒	☐	Antibodies
☒	☐	Eukaryotic cell lines
☐	☒	Palaeontology and archaeology
☒	☐	Animals and other organisms
☒	☐	Human research participants
☒	☐	Clinical data
☒	☐	Dual use research of concern

Methods

n/a	Involved in the study	
☒	☐	ChIP-seq
☒	☐	Flow cytometry
☒	☐	MRI-based neuroimaging

Palaeontology and Archaeology

Specimen provenance	No specimens were used. All data is available in the electronic supplement
Specimen deposition	No specimens were deposited. All data is available in the electronic supplement
Dating methods	No novel dates are produced. Occurrence ages, however, were updated to the latest chronostratigraphic standards based on the primary literature

☒ Tick this box to confirm that the raw and calibrated dates are available in the paper or in Supplementary Information.

Ethics oversight	No ethical guidance or approval was needed as all data is publically available

Note that full information on the approval of the study protocol must also be provided in the manuscript.
